# The natural compound gracillin exerts potent antitumor activity by targeting mitochondrial complex II

**DOI:** 10.1038/s41419-019-2041-z

**Published:** 2019-10-24

**Authors:** Hye-Young Min, Hyun-Ji Jang, Kwan Hee Park, Seung Yeob Hyun, So Jung Park, Ji Hye Kim, Jaekyoung Son, Sam Sik Kang, Ho-Young Lee

**Affiliations:** 1 Creative Research Initiative Center for Concurrent Control of Emphysema and Lung Cancer, College of Pharmacy, Seoul National University, Seoul, 08826 Republic of Korea; 2 Department of Molecular Medicine and Biopharmaceutical Sciences, Graduate School of Convergence Science and Technology and College of Pharmacy, Seoul National University, Seoul, 08826 Republic of Korea; 3 College of Pharmacy and Research Institute of Pharmaceutical Sciences, Seoul National University, Seoul, 08826 Republic of Korea; 4 Department of Biomedical Sciences, Asan Medical Center, AMIST, University of Ulsan College of Medicine, Seoul, 05505 Republic of Korea

**Keywords:** Drug development, Natural products, Phenotypic screening

## Abstract

Mitochondria play a pivotal role in cancer bioenergetics and are considered a potential target for anticancer therapy. Considering the limited efficacy and toxicity of currently available mitochondria-targeting agents, it is necessary to develop effective mitochondria-targeting anticancer drugs. By screening a large chemical library consisting of natural products with diverse chemical entities, we identified gracillin, a steroidal saponin, as a mitochondria-targeting antitumor drug. Gracillin displayed broad-spectrum inhibitory effects on the viability of a large panel of human cancer cell lines, including those carrying acquired resistance to chemotherapy or EGFR-targeting drugs, by inducing apoptosis. We show that gracillin attenuates mitochondria-mediated cellular bioenergetics by suppressing ATP synthesis and by producing reactive oxygen species (ROS). Mechanistically, gracillin disrupts complex II (CII) function by abrogating succinate dehydrogenase (SDH) activity without affecting the succinate:ubiquinone reductase. The gracillin-induced cell death was potentiated by 3-nitropropionic acid (3-NPA) or thenoyltrifluoroacetone (TTFA), which inhibit CII by binding to the active site of SDHA or to the ubiquinone-binding site, respectively. Finally, we show that gracillin effectively suppressed the mutant-*Kras*-driven lung tumorigenesis and the growth of xenograft tumors derived from cell lines or patient tissues. Gracillin displayed no obvious pathophysiological features in mice. Collectively, gracillin has potential as a CII-targeting antitumor drug.

## Introduction

Cancer is one of the leading causes of human death worldwide, mostly due to the genetic and molecular heterogeneity of cancer cells and the consequent marginal response to currently available anticancer therapies^1,2^. Therefore, there is a robust motivation to develop novel strategies for anticancer therapy. Cancer cells are known to face an insufficiency of oxygen and nutrients and exhibit a high demand for building blocks to support their aggressive proliferation and metastatic dissemination^3,4^. Accordingly, most cancer cells exhibit metabolic reprogramming, such as aerobic glycolysis, to support their aggressive proliferation and metastasis^5^. Moreover, altered metabolism enables cancer cells to be resistant to anticancer therapeutics^6^. Hence, targeting cancer metabolism has been proposed as an effective anticancer strategy. Indeed, preclinical and clinical studies have shown the potential of metabolism-targeting drugs, such as inhibitors of glycolysis, for anticancer treatment^7^. However, the clinical benefits of such metabolic modulators are unclear.

Recent evidence reveals that increased glycolysis does not occur in all tumors, and several cancer cells use mitochondrial respiration more than glycolysis^8–10^. Indeed, some mitochondrial metabolites, including reactive oxygen species (ROS) and succinate, activate oncogenic signal transduction and induce malignant transformation, and cancer cells utilize mitochondria for energy production, resulting in metabolic plasticity in cancer cells^11^. Alterations in oncogenes and tumor suppressor genes, as well as mutations in several tricarboxylic acid (TCA) cycle-associated enzymes and mitochondrial DNA are known to support mitochondrial metabolism^9,11^. Furthermore, depletion of mitochondrial DNA has been found to decrease tumorigenic potential^12^. Thus, modulation of mitochondrial metabolism has been proposed as a novel approach for anticancer therapy^9^. Indeed, previous preclinical and clinical studies have shown the anticancer potential of several mitochondrial modulators, including mitochondrial complex I inhibitors, mitochondrial ROS generators, and mitochondrial membrane potential disruptors^13^. However, the clinical use of these modulators is limited due to severe side effects, such as lactic acidosis and parkinsonism-like symptoms. Thus, it is necessary to discover novel anticancer mitochondrial modulators with diverse chemical structures and limited toxicity.

Several natural products, in particular, phytochemicals, have been identified as sources for the development of anticancer agents^14^. Their mechanisms of action vary greatly; however, many of them suppress cancer cell growth, and a few also modulate metabolism. To discover novel potential anticancer compounds targeting mitochondrial metabolism in cancer cells, we employed a large natural chemical library consisting of compounds from various chemical classes. By screening compounds to inhibit mitochondrial function in lung cancer cells, a major type of cancer cell known to exhibit metabolic heterogeneity individually^15^, we identified gracillin, a natural steroidal saponin that is a diosgenin glycoside, as a potential mitochondria-targeting antitumor agent in vitro and in vivo. Our findings showed that gracillin disrupted complex II-mediated mitochondrial function, leading to decreases in mitochondrial membrane potential, oxidative phosphorylation, and ATP synthesis and increases in mitochondrial ROS production and apoptotic cell death in cancer cells. Consistent with the in vitro inhibitory effects, gracillin displayed potent antitumor effects in vivo with a limited toxicity. These results suggest the potential of gracillin as an antitumor agent targeting mitochondrial respiration.

## Results

### Identification of gracillin, an active principle to target mitochondrial function in cancer cells

**Fig. 1:**
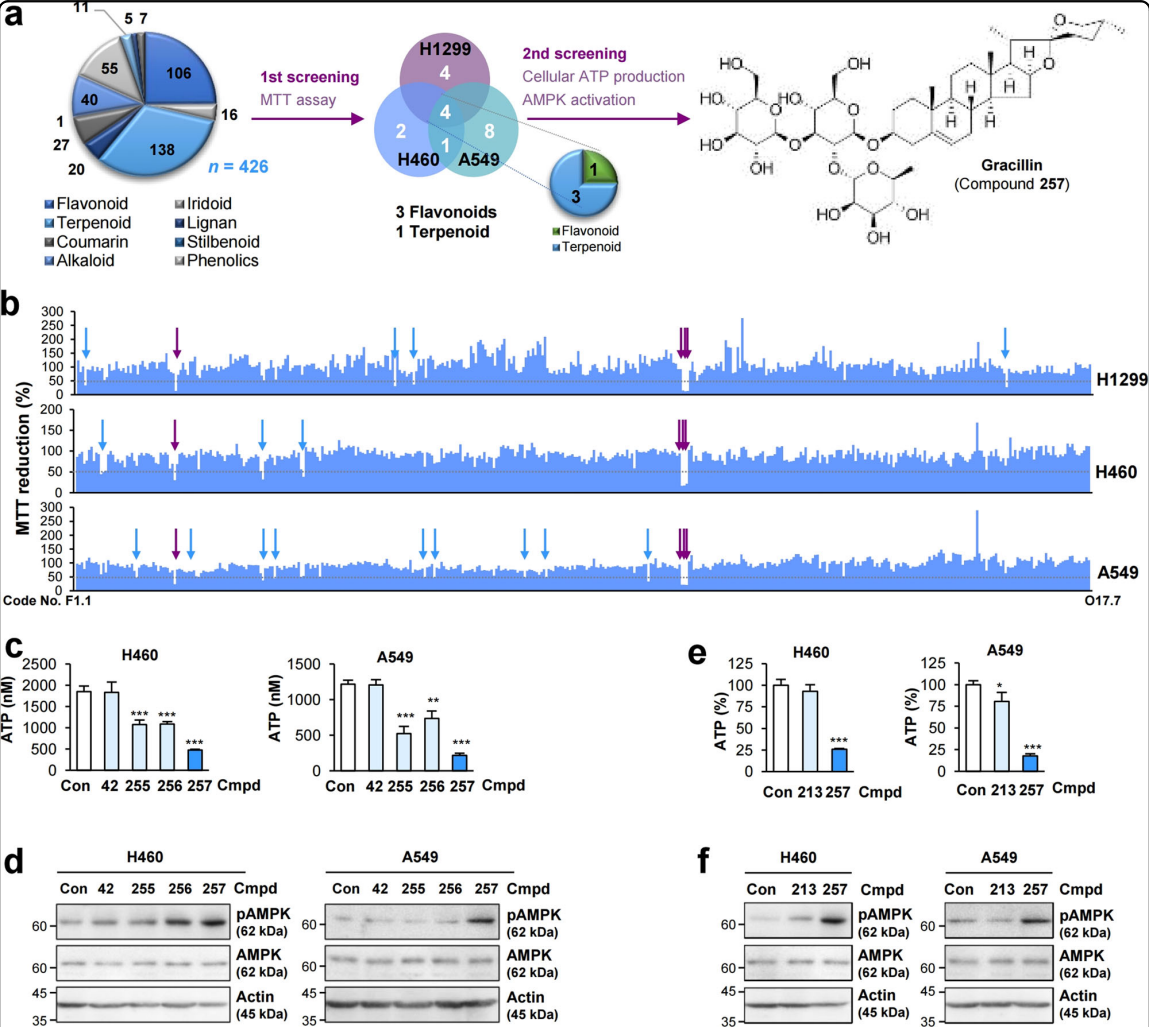
Identification of gracillin as an active compound to inhibit lung cancer cell viability by disrupting mitochondrial function. **a** Schematic diagram of the discovery of active compounds that inhibit cancer cell viability by targeting mitochondrial bioenergetics from the natural products-derived chemical library. **b** Effects of 426 compounds (10 μM) on the MTT-reducing ability (as an indication of cell viability) of H1299, H460, and A549 cells. Blue arrows indicate the active compounds that inhibit MTT reduction (compared with the vehicle-treated control group) by more than 50% in each cell line (H1299: *n* = 6; A549 and H460: *n* = 3). Purple arrows indicate the compounds that are active in all three cell lines. **c**, **e** The effect of test compounds (5 μM) on the cellular ATP level was determined by using a luminescence-based ATP assay kit (*n* = 5). **e** The percentage of ATP level in cells treated with test compounds was determined by comparison with the vehicle-treated control group. **d**, **f** The effects of the test compounds on the activation of AMPK were determined by Western blot analysis. Bottom. Densitometric analysis of phosphorylated AMPK levels by comparison with total AMPK levels was performed by using ImageJ software (*n* = 3). The bars represent the mean ± SD; *P < 0.05, **P < 0.01, and ***P < 0.001, as determined by a two-tailed Student’s *t*-test compared with the vehicle-treated control group. Con Control, Cmpd Compound, GRA gracillin

To discover anticancer drugs that target mitochondrial metabolism, we screened a large natural-product chemical library consisting of 426 compounds with various chemical classes (Fig. 1a) by performing the MTT assay, which detects mitochondrial dehydrogenase activity and has been used for determining mitochondrial function^16^. We identified four most potent compounds that consistently induced MTT reduction by more than 50% in three NSCLC cell lines (H1299, H460, and A549) (Fig. 1b**;** Supplementary Table 1**;** Supplementary Fig. 1). These compounds were a flavonoid (Compound **42**) and three steroidal saponins (diosgenin glycosides) (Compounds **255**, **256**, and **257**). We next examined the effect of these active compounds on cellular energetics, including production of ATP and activation (phosphorylation) of AMP-activated protein kinase (AMPK), a cellular energy sensor^17^. Among the diosgenin glycosides, gracillin (Compound **257**) (Fig. 1a) displayed the most profound effects on suppressing ATP production (Fig. 1c) and subsequently inducing AMPK phosphorylation (Fig. 1d). Because a glycoside moiety in gracillin is associated with various biological activities^18^, we compared the ability of gracillin with that of its aglycone diosgenin (Compound **213**) to exert an inhibitory effect on cellular energetics. Gracillin showed greater activities in suppressing ATP production (Fig. 1e) and activating AMPK (Fig. 1f) than did diosgenin. These findings suggested that the glycoside moiety is essential for the capacity of gracillin to regulate cellular ATP production.

**Fig. 2:**
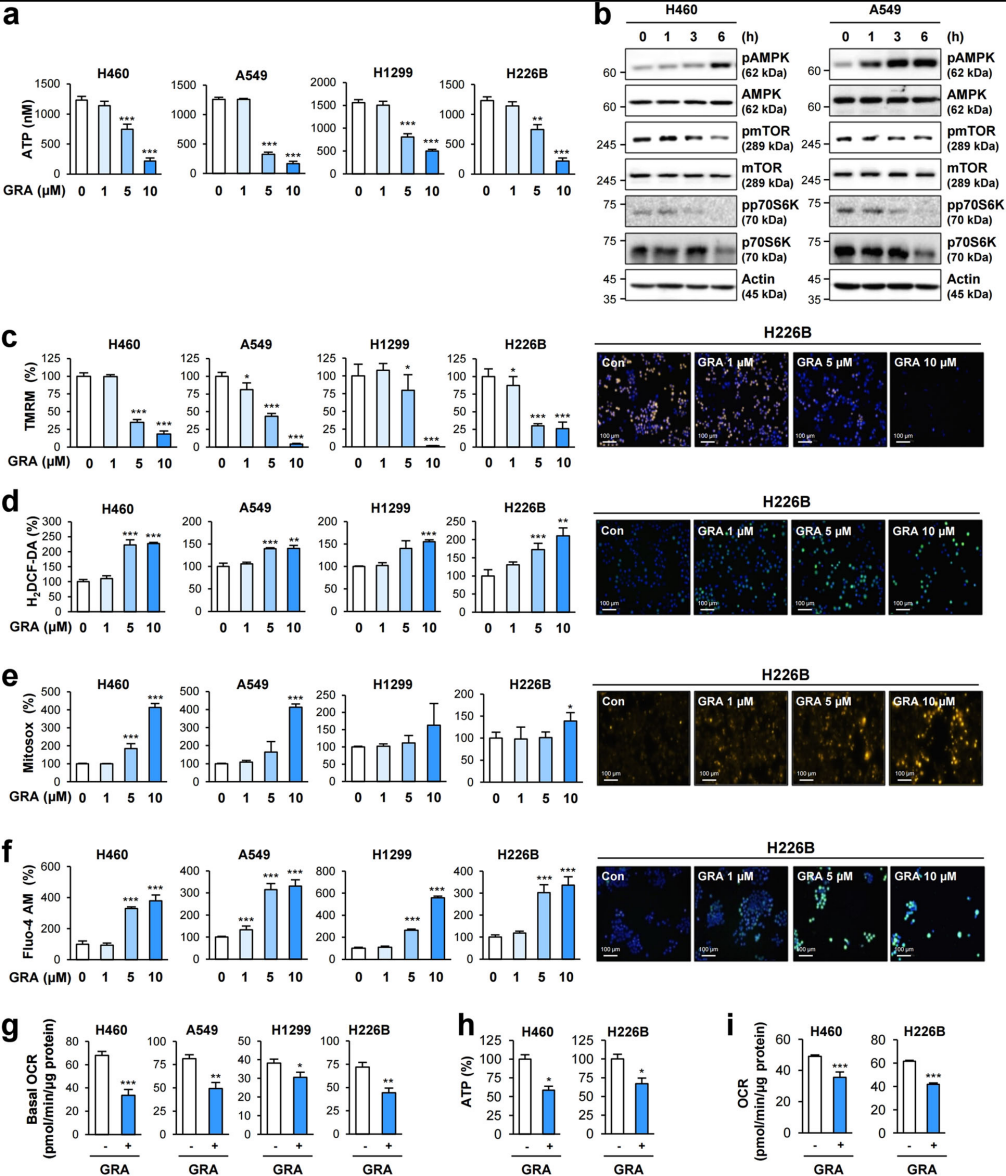
Inhibitory effect of gracillin on cellular ATP production through inducing mitochondrial dysfunction. **a** Dose-dependent effects of gracillin on the cellular ATP level were determined by using a luminescence-based ATP assay kit (*n* = 5). **b** Time-dependent modulation of AMPK and its downstream signaling by treatment with gracillin (5 μM) was examined by Western blot analysis. **c**–**f** NSCLC cells were treated with increasing concentrations (0, 1, 5, and 10 μM) of gracillin for 6 h. Cells were stained with TMRM (**c**), H_2_DCF-DA (**d**), Mitosox (**e**), and fluo-4 AM (**f**) dyes and analyzed as described in the Materials and Methods section (*n* = 4). Right. Representative images of H226B cells stained with TMRM, H_2_DCF-DA, Mitosox, and fluo-4 AM. **g** The effect of gracillin (5 μM) on basal OCRs in NSCLC cells was determined using a Seahorse XF analyzer (*n* = 3). **h**, **i** The effects of gracillin on ATP production (**h**) and basal OCRs (**i**) in isolated mitochondria were determined by a luminescence-based ATP assay kit and Seahorse XF analyzer, respectively (*n* = 3). The bars represent the mean ± SD; *P < 0.05, **P < 0.01, and ***P < 0.001, as determined by a two-tailed Student’s *t*-test by comparison with the vehicle-treated control group. GRA: gracillin

We confirmed dose-dependent suppression of ATP production by the gracillin treatment in a subset of NSCLC cell lines (Fig. 2a). The gracillin-induced increase in phosphorylation of AMPK and inactivation of downstream effectors of AMPK, including mTOR and p70S6K, occurred as early as 1 h after the treatment (Fig. 2b). We next determined the effects of gracillin on mitochondrial function. A fluorescence probe that specifically detect functional mitochondria [tetramethylrhodamine methyl ester (TMRM)]^19^ revealed a dose-dependent decrease in TMRM pixel intensity after the gracillin treatment (Fig. 2c). Because deregulated mitochondrial function leads to ROS generation^20^ and ROS further disrupts mitochondrial function^21^, we examined whether gracillin treatment subsequently generate mitochondrial ROS. We observed dose-dependent increases in intracellular ROS (H_2_DCF-DA)^22^ (Fig. 2d) and mitochondrial superoxide (Mitosox)^23^ (Fig. 2e) in the gracillin-treated cells. Intracellular calcium, which enhances ROS production when respiratory complexes are inhibited pharmacologically^24^, was also elevated by treatment with gracillin (Fig. 2f). When cellular oxygen consumption was analyzed using the Seahorse extracellular flux analyzer, gracillin treatment induced dose-dependent decreases in the OCR, an indicator of mitochondrial respiration^25^ (Fig. 2g). Importantly, gracillin significantly suppressed the ATP production (Fig. 2h) and oxygen consumption rate (OCR) (Fig. 2i) in isolated mitochondria. These results clearly indicated that the gracillin-induced inhibition of cellular energetics was caused at least in part by disruption of mitochondrial function.

### Disruption of mitochondrial function by gracillin treatment leads to the suppression of cancer cell viability by inducing apoptosis

**Fig. 3:**
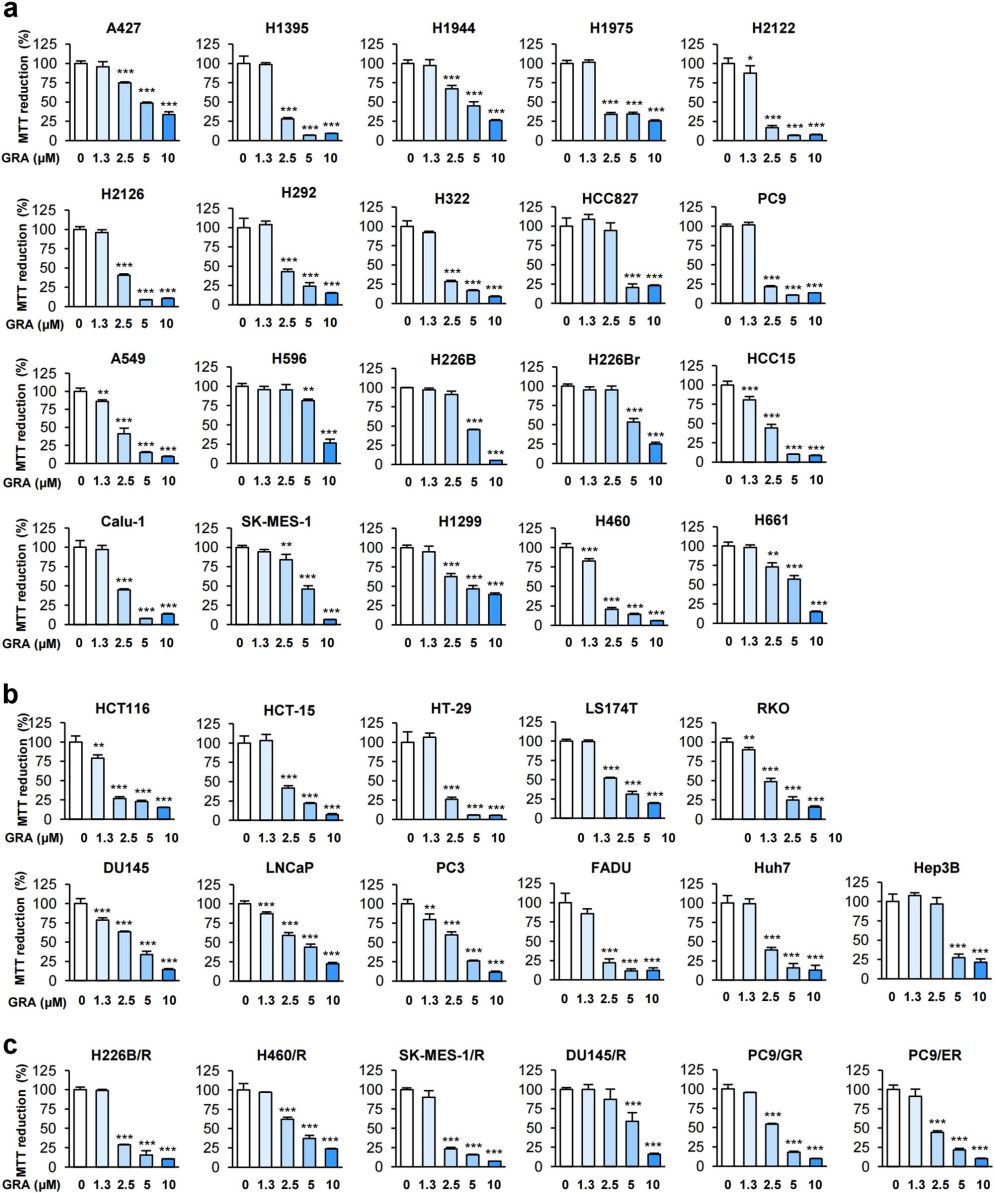
Inhibitory effects of gracillin on the mitochondrial activity of various cancer cells. (**a**–**c**) An MTT assay was used to determine the effects of gracillin on the mitochondrial activity (an indicator of cell viability) of various cancer cells originating from the lung (**a**) or other organs (**b**; prostate: DU145, LNCaP, PC3; pharynx: FADU; colorectum: HCT-15, HCT116, HT-29, LS174T, RKO; liver: Huh7, Hep3B), as well as of cancer cells carrying acquired resistance to paclitaxel (H226B/R, H460/R, SK-MES-1/R, and DU145/R), gefitinib (PC9/GR), or erlotinib (PC9/ER) (**c**) (*n* = 4). The bars represent the mean ± SD; *P < 0.05, **P < 0.01, and ***P < 0.001, as determined by a two-tailed Student’s *t*-test by comparison with the vehicle-treated control group. GRA gracillin

We next assessed the capacity of gracillin to inhibit mitochondrial function (which is correlated with cell viability) in a large panel of human cancer cell lines. The MTT assay displayed dose-dependent inhibitory effects of gracillin on the mitochondrial function in various human cancer cell lines derived from lung, colorectum, prostate, pharynx, and liver with IC_50_ values of ~1–5 μM (Supplementary Table 2**;** Fig. 3a, b). Gracillin also displayed substantial inhibitory effects on the mitochondrial function of several cancer cell sublines that had acquired resistance to anticancer therapeutics (designated ‘/R’), including the chemotherapeutic drug paclitaxel (H226B/R, H460/R, SK-MES-1/R, DU145/R) and the EGFR tyrosine kinase inhibitors (TKIs) gefitinib (PC9/GR) or erlotinib (PC9/ER) (Supplementary Table 2**;** Fig. 3c). These results indicated the potential of gracillin as a mitochondria-targeting agent.

**Fig. 4:**
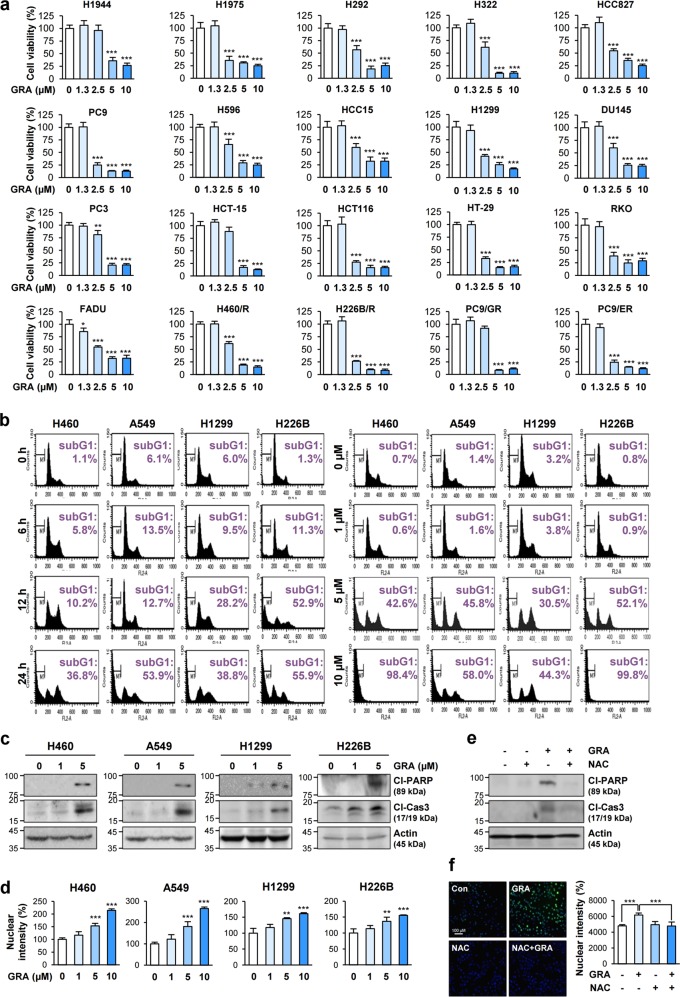
Inhibitory effects of gracillin on the viability of various cancer cells by inducing apoptosis. **a** Effects of gracillin on the viability of various cancer cells were determined by a crystal violet assay (*n* = 6). **b** H460, A549, H1299, and H226B cells were treated with 5 μM gracillin for 6, 12, or 24 h (left) or with various concentrations (0, 1, 5, and 10 μM) of gracillin for 24 h. Analysis of the cell cycle distribution using PI-stained samples was performed by flow cytometry. **c** NSCLC cells were treated with increasing concentrations (0, 1, and 5 μM) of gracillin for 24 h. The cleavage of PARP (Cl-PARP) and caspase-3 (Cl-Cas3) was determined by Western blot analysis. **d** NSCLC cells were treated with increasing concentrations (0, 1, 5, and 10 μM) of gracillin for 6 h. Cells were stained with Hoechst 33342 (1 μg/ml). Nuclear intensity was analyzed by using an Operetta high content imaging system (*n* = 6). **e**, **f** Abrogation of gracillin-induced apoptosis and intracellular ROS production by treatment with NAC (10 mM) in combination. A549 cells were treated with gracillin (5 μM) alone or in combination with NAC (10 mM) for 6 (**f**) or 24 (**e**) hours. **e** The cleavage of PARP (Cl-PARP) and caspase-3 (Cl-Cas3) was determined by Western blot analysis. **f** Left. The level of intracellular ROS was determined after staining cells with 20 μM H_2_DCF-DA. Right. The level of chromatin condensation was determined by analysis of the nuclear intensity of Hoechst 33342-stained cells using an Operetta High Content Imaging System (*n* = 6). The bars represent the mean ± SD; *P < 0.05, **P < 0.01, and ***P < 0.001, as determined by a two-tailed Student’s *t*-test by comparison with the vehicle-treated control group (**a, d**) or one-way analysis of variance (ANOVA) with Tukey’s multiple comparisons test (**f**). GRA gracillin

We next assessed the effects of gracillin on cancer cell viability by performing the crystal violet assay. Gracillin treatment exhibited broad-spectrum inhibitory effects on the viability of various types of human cancer cells (Fig. 4a). However, in contrast to the minimal effect of gracillin on the viability of HT-29 cells in the previous literature^26^, we observed that gracillin treatment significantly suppressed the viability of HT-29 cells. This discrepancy might be due to the differences in the assay systems used in the two studies. The sulforhodamine B (SRB) used in the previous study binds to proteins under mild acidic conditions^27^, whereas crystal violet used in this study stains both protein and DNA^28^. The experimental conditions, such as the number of inoculated cells (i.e., 5000 cells per well used in the previous study according to the National Cancer Institute anticancer screening system^29^ vs. 2000 cells per well in our study), the duration of drug treatment (i.e., 48 h in the previous study vs. 72 h in our study), and other technical differences between the two studies might also cause the difference in the gracillin sensitivity of HT-29 cells. Cell cycle analysis revealed that gracillin increased the number of NSCLC cells in the sub-G1 phase in time-dependent and dose-dependent manners (Fig. 4b). Gracillin-treated cancer cells also showed cleavage of both poly (ADP-ribose) polymerase (PARP) and caspase-3 (Fig. 4c) and dose-dependent increases in chromatin condensation (Fig. 4d). Considering the close association between mitochondrial activity and cell viability^16^, the gracillin-mediated effects against mitochondrial function seemed to suppress viability of various types of cancer cells, including chemoresistant and EGFR TKI-resistant cancer cells, by inducing apoptosis. Moreover, treatment with an antioxidant N-acetyl-l-cysteine (NAC) in combination almost completely abolished the gracillin-mediated effects, such as PARP and caspase-3 cleavages, chromatin condensation, and inhibition of mitochondrial function (Fig. 4e, f; Supplementary Fig. 2). Under the same experimental conditions, treatment with NAC in combination markedly suppressed gracillin-induced intracellular ROS production (Fig. 4f). These results collectively suggest that oxidative stress caused by gracillin-induced disruption of mitochondrial function is crucial for the cytotoxic and proapoptotic effects of gracillin in NSCLC cells.

### Gracillin inhibits mitochondria-mediated cellular bioenergetics by disrupting mitochondrial complex II (CII) function

**Fig. 5:**
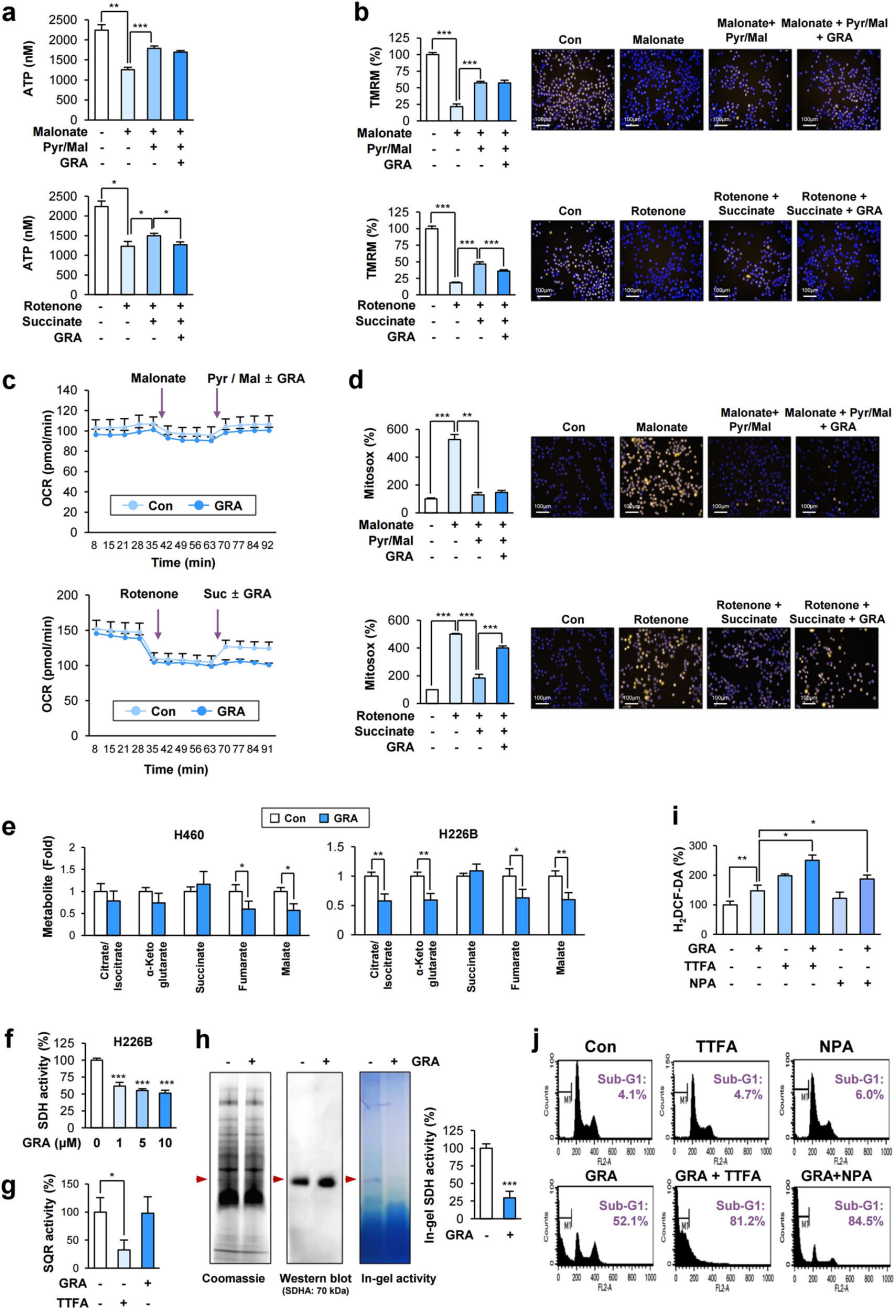
Inhibition of mitochondrial CII activity by treatment with gracillin. **a**–**d** H226B cells were preincubated with sodium malonate (2 mM) or rotenone (1 μM) to suppress CII [complex II; for measurement of complex I (CI) activity] or CI (for measurement of CII activity), respectively. Thereafter, the cells were stimulated with substrates for CI [pyruvate (5 mM) and malate (2.5 mM)] or CII [succinate (10 mM)] in the presence or absence of gracillin (5 μM). **a** After 6 h, cellular ATP levels were determined by using an ATP lite ATP assay kit (*n* = 4). **b** After 6 h, cells were further stained with TMRM dye and analyzed by an Operetta high content imaging system (*n* = 8). **c** OCRs were determined using a Seahorse XF analyzer (*n* = 3). **d** After 6 h, cells were further stained with Mitosox and analyzed by using an Operetta high content imaging system (*n* = 10). **e** Cells were treated with gracillin (5 μM) for 8 h. Metabolic changes associated with the TCA cycle in vehicle-treated and gracillin-treated NSCLC cells (*n* = 3). **f**, **g** The effects of gracillin on SDH (*n* = 4) (**f**) or SQR (*n* = 3) (**g**) activity was determined as described in the Materials and Methods section. **g** TTFA was used as a positive control. **h** Effects of gracillin on the assembly of CII and the SDH activity were determined by BN-PAGE of mitochondrial extracts with or without gracillin treatment, followed by visualization with Coomassie blue staining (left), Western blot analysis to detect a CII marker SDHA (middle), and in-gel SDH activity assay (right), as described in Materials and Methods section. Red arrows indicated bands with the CII complex and the SDH activity. The graph depicts densitometric analysis of the band in in-gel SDH activity (*n* = 3). **i, j** Enhancement of gracillin-induced ROS generation (**i**) and apoptosis (**j**) by treatment with TTFA (100 μM) or 3-NPA (NPA; 2 mM) in combination with gracillin (5 μM). H226B cell were treated with gracillin alone or in combination with TTFA or 3-NPA for 6 (**i**) or 24 (**j**) hours. The effects on gracillin-induced ROS generation (**i**) and apoptosis (**j**) was determined by fluorometric analysis after staining with 20 μM H_2_DCF-DA (Control and GRA: *n* = 6; Other groups: *n* = 3). **h** and flow cytometry (**i**), respectively, as described in Materials and Methods section. The bars represent the mean ± SD; *P < 0.05, **P < 0.01, and ***P < 0.001, as determined by a two-tailed Student’s *t*-test (**e, f, g, h**), one-way ANOVA with Tukey’s multiple comparisons test (**a**), Kruskal-Wallis test with Dunn’s multiple comparisons test [**5b** (top), **5d** (top)], or Brown-Forsythe and Welch ANOVA tests with Dunnett’s T3 multiple comparisons test [**5b** (bottom), **5d** (bottom), **i**]. Con control, GRA gracillin

We investigated the mechanism underlying the gracillin-induced disruption of mitochondrial function. Since electrons in the respiratory chain are transferred from complex I (CI) and CII to complex III (CIII)^30,31^, we determined the specificity of the gracillin-mediated mitochondrial dysfunction. We measured mitochondrial function in H226B cells wherein either CI was stimulated with pyruvate/malate after pretreatment with the CII inhibitor malonate^30^ or CII was stimulated with succinate after pretreatment with the CI inhibitor rotenone^32^. Exposure to each complex-specific substrate elevated the ATP synthesis (Fig. 5a), TMRM intensity (Fig. 5b), OCR (Fig. 5c), and mitochondrial ROS production (Fig. 5d). Notably, the CII substrate-induced events were significantly suppressed by the gracillin treatment, while CI substrate-induced events were minimally affected (Fig. 5a-d), suggesting the capacity of gracillin to abrogate the CII-specific mitochondrial function.

To further assess the capacity of gracillin to inhibit CII function, we identified the metabolites in gracillin-treated NSCLC cells by performing a liquid chromatography/mass spectrometry (LC/MS) analysis. As shown in Fig. 5e, the level of succinic acid was moderately increased in gracillin-treated NSCLC cells, whereas the levels of citrate/isocitrate, α-ketoglutarate, fumarate, and malate were substantially decreased by treatment with gracillin. The significant downregulation of fumarate and malate in two gracillin-treated cells suggested the inhibitory effect of gracillin on SDH activity. As expected, treatment with gracillin significantly decreased the SDH activity as determined by the MTT reduction^33^ (Fig. 5f; Supplementary Fig. 3). We further measured the effects of gracillin on the succinate:ubiquinone oxidoreductase (SQR) activity by utilizing 2,6-dichloroindophenol (an electron acceptor) and decylubiquinone. Compared with TTFA that is known to abrogate CII function by inhibiting SQR activity, gracillin minimally affected SQR activity (Fig. 5g). These results suggest that inhibition of SDH activity is involved in the gracillin-mediated CII inhibition.

Mitochondrial CII comprises four subunits (SDHA, SDHB, SDHC, and SDHA) and exerts SDH activity as a component of the TCA cycle and SQR activity as a component of the respiratory chain^10,34^. We determined whether gracillin-mediated abrogation of CII function was associated with changes in the expression of the four subunits. Protein expression of these subunits remained minimally changed after the gracillin treatment in two representative NSCLC cell lines (Supplementary Fig. 4a). Moreover, the association of SDHA with other CII subunits was minimally altered by gracillin treatment (Supplementary Fig. 4b). Pull-down assays using recombinant SDHA (His-tagged SDHA) and NSCLC cell lysates further confirmed the minimal impact of gracillin treatment on the assembly of CII subunits (Supplementary Fig. 4c). We further evaluated the effects of gracillin treatment on the assembly of the complex in native conditions. Blue Native PAGE (BN-PAGE)^35,36^ and subsequent in-gel enzyme activity assay using mitochondrial extracts showed that gracillin had minimal impact on the association of CII subunits (Fig. 5h). In contrast, the in-gel SDH activity was markedly suppressed by treatment with gracillin, suggesting that gracillin suppresses the function of mitochondrial complex II without affecting the assembly of the complex II subunits (Fig. 5h). Therefore, gracillin might regulate SDH activity through a mechanism other than inhibiting the expression or assembly of CII subunits.

We next tested the effects of combined treatment with gracillin plus TTFA, a CII inhibitor that binds to the ubiquinone-binding site of CII^37^, or 3-NPA, a CII inhibitor that suppresses the electron transfer from succinate to FAD by interacting with the active site of SDHA^38^, on apoptotic activities and ROS generation. Consistent with the previously published findings^38^, treatment with TTFA alone significantly increased ROS production while ROS levels were minimally increased in NPA-treated cells. (Fig. 5i). Notably, TTFA or 3-NPA combination enhanced gracillin-mediated apoptotic cell death in a similar level while combination with TTFA enhanced gracillin-mediated ROS generation greater than 3-NPA combination (Fig. 5i, j). The potentiation of gracillin-induced apoptosis by TTFA might be due to the complete blockade of CII function by suppression of both SDH and SQR activity. Considering various cellular targets of 3-NPA other than SDHA^39^, the potentiation of gracillin-induced apoptosis by NPA might be through additional mechanisms other than SDH. These results also suggested that gracillin might interact with SDH and inhibit the CII function with a mode of action different from TTFA and 3-NPA, thereby inducing apoptotic death in cancer cells.

### Antitumor effect of gracillin on the growth of NSCLC cell-line-derived and patient-derived xenograft tumors and mutant-*Kras*-driven spontaneous lung tumors with minimal toxicity in vivo

**Fig. 6:**
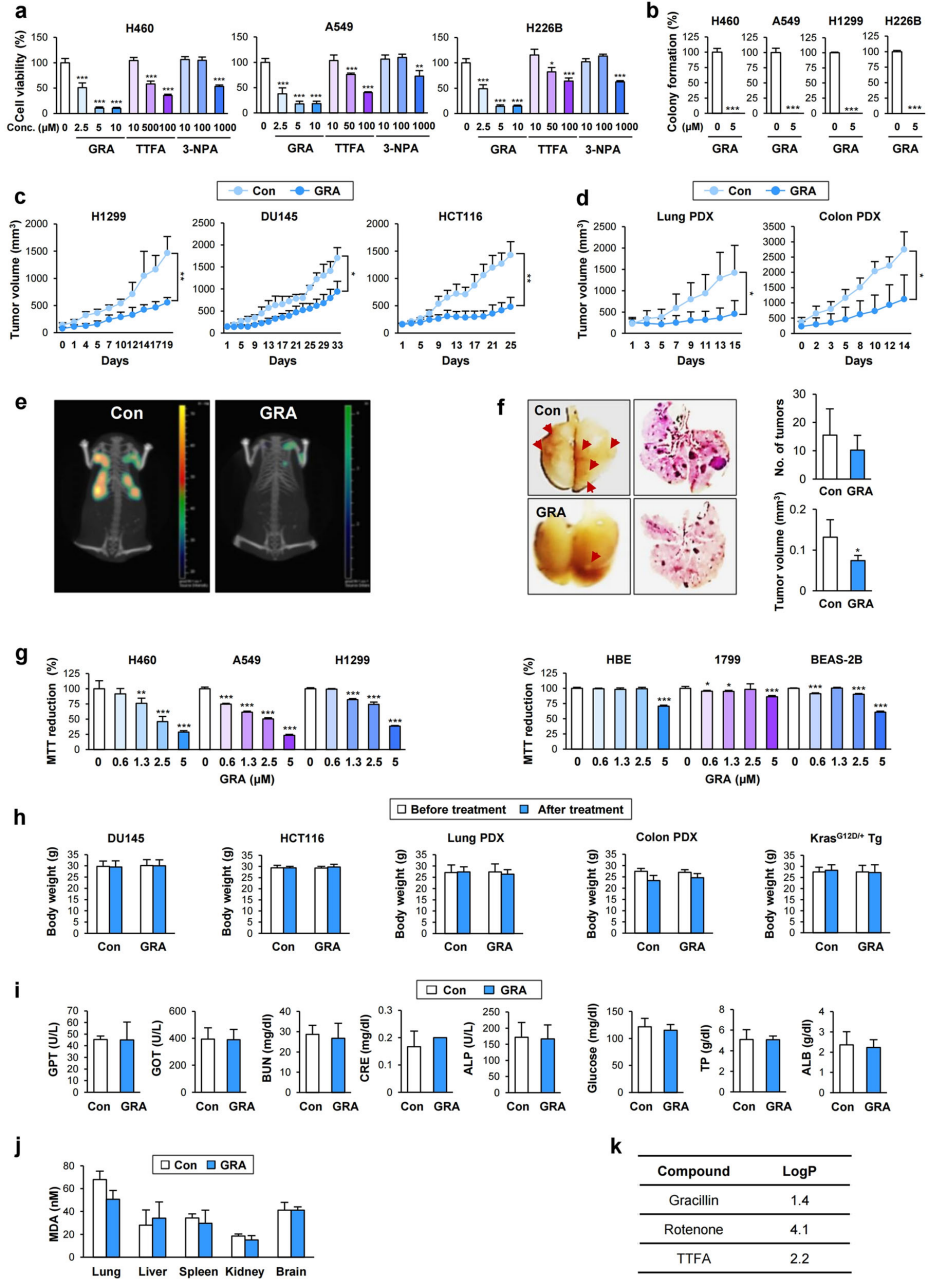
Inhibitory effects of gracillin on the colony formation of NSCLC cells in vitro and tumor growth in vivo with limited toxicity. **a** The superior inhibitory effects of gracillin on the viability of NSCLC cells by comparison with known CII inhibitors (TTFA and 3-NPA) were determined by a crystal violet assay (*n* = 4). **b** The inhibitory effects of gracillin on clonogenicity under anchorage-dependent culture conditions were determined by an anchorage-dependent colony formation assay (*n* = 3). **c**, **d** Antitumor effects of gracillin in tumor xenograft models inoculating various cancer cells [H1299 (Con: *n* *=* 4, GRA: *n* = 6); DU145 (Con: *n* *=* 4, GRA: *n* = 4); HCT116 (Con: *n* *=* 4, GRA: *n* = 6)] (**c**) and patient-derived tumor tissues [Lung PDX (Con: *n* *=* 6, GRA: *n* = 6); Colon PDX (Con: *n* *=* 4, GRA: *n* = 10)] (**d**). **e**, **f** Decrease in mutant-*Kras*-driven lung tumorigenesis by treatment with gracillin (Con: *n* *=* 4, GRA: *n* = 4) was determined by IVIS imaging using an MMPSense 680 probe (**e**) and by the observation of H&E-stained tissues (**f**, left). Representative images are shown. (**f**, right) Microscopic evaluations of lung tissue to measure the mean tumor number and volume using H&E-stained tissues were performed as described in the Materials and Methods section (*n* = 4). **g** NSCLC (H460, A549, and H1299) and normal HBE (HBE, 1799, and BEAS-2B) cell lines were treated with various concentrations of gracillin for 24 h. Changes in the mitochondrial activity (an indicator of cell viability) were determined by the MTT assay (*n* = 5). **h** Body weight changes in vehicle-treated or gracillin-treated mice (*n* = 4–10, as indicated in **c** and **d**). **i** The levels of glutamic oxaloacetic transaminase (GOT), blood urea nitrogen (BUN), creatinine, alanine aminotransferase (GPT), alkaline phosphatase (ALP), glucose, total protein (TP), and albumin (ALB) in the serum from vehicle-treated or gracillin-treated mutant-*Kras* transgenic mice (*n* = 4). **j** The level of lipid peroxidation, as indicated by the level of malonaldehyde (MDA), in various tissues (lung, liver, spleen, kidney, and brain) obtained from mutant-*Kras* transgenic mice treated with vehicle or gracillin (*n* = 4). **k** The LogP value of each compound calculated by the XLogP3 or the XLogP3-AA methods, as an indicator of BBB permeability, was obtained from PubChem (http://pubchem.ncbi.nlm.nih.gov/). The bars represent the mean ± SD; *P < 0.05, **P < 0.01, and ***P < 0.001, as determined by a two-tailed Student’s *t*-test (**a**, **b**, **g**) or Mann-Whitney test (**c**, **d**, **f**) by comparison with the vehicle-treated control group. Con control, GRA gracillin

We investigated the potential of gracillin as a mitochondria-targeting antitumor agent. Firstly, we compared the cytotoxic potency of gracillin with that of known CII inhibitors. As shown in Fig. 6a, the inhibitory effects of gracillin on several NSCLC cell viability was superior to that of other CII inhibitors, such as TTFA and 3-NPA. Clonogenic survival of NSCLC cells were also significantly suppressed by the gracillin treatment (Fig. 6b). Because conventional cancer cell lines can adapt to artificial in vitro culture conditions and the interaction of tumor cells with stromal components supports cancer cell survival^40^, we evaluated antitumor activities of gracillin in vivo in xenograft models of various cancer cell lines. We found that oral gavage treatment with gracillin significantly reduced the growth of xenograft tumors of lung (H1299), prostate (DU145), and colorectum (HCT116) cancer cells (Fig. 6c). These findings indicated the potential of gracillin as a potent antitumor agent applicable to tumors originating from various types of organs. To assure the clinical applicability of gracillin, we further addressed the effects of gracillin on the growth of patient-derived xenograft (PDX) tumors and found potent inhibitory effects on their growth (Fig. 6d).

We next assessed whether gracillin inhibits lung tumor growth in vivo using *Kras* transgenic mice that develop spontaneous lung tumors with a 100% incidence^41^. Differences in tumor growth were monitored by fluorescence-based image analyses. A representative mouse showed markedly reduced lung tumor growth by gracillin treatment (Fig. 6e). Postmortem examination of the mice revealed that gracillin-treated mice had fewer lung tumor nodules than vehicle-treated mice (Fig. 6f). Microscopic analysis of hematoxylin and eosin (H&E)-stained lung tissues revealed substantial decreases in the tumor multiplicity and volume in gracillin-treated mice compared with the control mice. (Fig. 6f) These data indicated the significant inhibitory effect of gracillin on mutant-*Kras*-driven lung tumor growth.

We finally examined the toxicity of gracillin. We tested the effects of gracillin on a panel of NSCLC (H460, A549, and H1299) and immortalized normal lung epithelial (HBE, 1799, and BEAS-2B) cell lines within the same range of concentrations. In contrast to the dose-dependent decreases in the mitochondrial function of NSCLC cells, the gracillin treatment had minimal impact on the mitochondrial function of the normal lung epithelial cells. These results may exclude major mitochondrial toxicity of gracillin in normal lung epithelial cells (Fig. 6g). In addition, the vehicle-treated and gracillin-treated mice in the three animal models (Fig. 6c-e) showed negligible difference in body weight (Fig. 6h). We further determined the serum levels of glutamate pyruvate transaminase (GPT) and glutamic oxaloacetate transaminase (GOT) (markers of liver toxicity), blood urea nitrogen (BUN), and creatinine (markers of kidney toxicity), alkaline phosphatase (ALP) (a marker of bile duct obstruction), blood glucose (a marker of metabolic disorder), and total protein (TP) and albumin (ALB) (markers of systemic inflammation) in vehicle-treated and gracillin-treated mutant-*Kras* mice to determine potential toxicities after long-term treatment with gracillin. As shown in Fig. 6i, the levels of these markers were not significantly different between vehicle-treated and gracillin-treated mice, indicating that gracillin displayed minimal toxic effects on the liver and kidney and did not cause metabolic disorders or system inflammation. Moreover, the level of lipid peroxidation, a marker of oxidative stress-mediated cellular injury^42^, in various organs including the lung, liver, spleen, kidney, and brain was not significantly changed by treatment with gracillin (Fig. 6j). Therefore, although gracillin induces cellular oxidative stress in cancer cells, gracillin may not cause oxidative stress-mediated tissue damage in normal tissues. Furthermore, an in silico prediction of blood-brain barrier (BBB) permeability^43^ suggested that gracillin may have low BBB permeability (Fig. 6k), indicating that gracillin may be less likely to induce the neurotoxicity that has been suggested as a toxic effect of SDH inhibitors^30^. These overall results suggest that gracillin has clinically applicable, efficient antitumor activities with minimal toxicities.

## Discussion

In this study, we aimed to discover mitochondria-targeting antitumor agents by screening a large natural product chemical library. We demonstrate herein gracillin as a mitochondria-targeting active principle that suppresses viability of a broad spectrum of cancer cell lines by inducing apoptosis. We further demonstrate the capacity of gracillin to suppress the growth of cell line-derived and patient-derived xenograft tumors and the mutant-*Kras*-driven spontaneous lung tumorigenesis in vivo without overt toxicity. We also performed in-depth mechanistic studies and demonstrate that gracillin suppresses mitochondrial CII function by abrogating SDH activity, leading to inhibition of ATP synthesis and production of ROS. These results suggest the potential of gracillin as a mitochondrial CII-targeting antitumor agent with minimal toxicity.

Metabolic reprogramming utilizing aerobic glycolysis as a main energy source has been considered a hallmark of cancer^44^. However, recent findings demonstrating functional mitochondria in cancer cells^8–10^ suggest that targeting mitochondria-mediated respiration can be a strategy for anticancer treatment. Considering the limited clinical use of mitochondria-targeting anticancer agents, it is necessary to develop safe and efficacious anticancer agents targeting mitochondrial function. In this regard, our study might be of importance to the development of mitochondria-targeting anticancer hits and/or lead candidates.

Firstly, our study identified a potential mitochondria-targeting antitumor agent by analyzing a large panel of compounds derived from natural products. Natural products are valuable sources for procurement of potential anticancer agents^14^. The value of natural products in drug discovery is due to their structural, functional, and mechanistic diversities^45^. Mitochondrial CII has been regarded as an attractive target in drug discovery based on its function as the branching point connecting the TCA cycle and electron respiratory chain and as the mediator of apoptosis^30^. Previous studies have identified various CII inhibitors, including vitamin D analogs, 3-bromopyruvate, and malonate^30^. In this study, we demonstrate gracillin as a new chemical entity functioning as a mitochondrial CII-targeting anticancer agent. Gracillin and other steroidal saponins have shown antitumor activities in several types of human cancer cells by inducing cell cycle arrest and apoptosis^26,46,47^. However, action mechanisms underlying the antitumor activities of gracillin have been elusive.

Our study shows that mitochondrial respiratory chain CII is a cellular target for the antitumor effect of gracillin. Interestingly, gracillin significantly suppresses ATP production and subsequent pAMPK (T172) levels in A549 and H460 cells carrying mutated *STK11* (LKB1)^48^ that is important for the phosphorylation of AMPK at T172 and subsequent its kinase activity. These results suggest that gracillin activates AMPK via other kinase, such as calmodulin-dependent protein kinase kinase-β (CaMKKβ)^49,50^ rather than LKB1, or via other mechanisms independent of mitochondrial complex II. We found that gracillin significantly elevated intracellular calcium and mitochondrial ROS in NSCLC cells. Since calcium is an activator of CaMKKβ, an upstream kinase for AMPK phosphorylation at T172^51^, calcium could have been involved in the gracillin-induced AMPK phosphorylation in A549 and H460 cells. Otherwise, gracillin-mediated elevation of cellular ROS could have induced depletion of intracellular energy through mitochondrial dysfunction^21^.

Our results show that gracillin directly targets the conversion of succinate into fumarate (SDH activity) rather than succinate:ubiquinone oxidoreductase (SQR). However, this inhibition does not lead to a significant accumulation of succinate in the cells. Based on our finding that gracillin induced substantial decrease in the level of citrate/isocitrate and α-ketoglutarate, we reasoned that the marginal accumulation of succinate in the gracillin-treated cells might be due to the decrease in the level of citrate/isocitrate and α-ketoglutarate, metabolic intermediates that are converted to succinate by sequential enzymatic reactions in the TCA cycle. These results also suggested that gracillin could have induced antitumor activities by regulating glycolysis-mediated cellular bioenergetics in addition to mitochondrial respiration. Although the precise mechanism by which gracillin inhibits CII needs to be investigated in further studies and additional studies to investigate whether gracillin can regulate glycolysis are ongoing, our results may contribute to better understanding of the action mechanisms of gracillin.

Secondly, our study provides gracillin as a novel mitochondria-targeting antitumor agent with clinical applicability and an improved safety profile. Previous studies have shown the antitumor properties of this compound^26,46,47^. In detail, gracillin exerted broad-spectrum cytotoxicity in various human cancer cell lines^26^, and gracillin induced cell cycle arrest at G1 phase, apoptosis, and cellular oxidative stress in HL-60 cells^47^. However, these findings were obtained from in vitro studies, and antitumor activities of gracillin have not been assessed in vivo systems. Our study reveals broad-spectrum antitumor activities of gracillin in vitro in various human cancer cell lines and in vivo in mutant-*Kras*-induced spontaneous lung tumors. We further show that gracillin markedly suppressed the growth of xenograft tumors of NSCLC and colon cancer cell lines and those of patients with lung and colon cancer. These findings clearly show the potential clinical applicability of gracillin. Major drawbacks of the clinical utilization of mitochondria-targeting anticancer agents are side effects and toxicity. In the case of CII inhibitors, inhibition of SDH activity may lead to several side effects in organs highly dependent on the TCA cycle and mitochondrial respiration, such as neurons and the heart^30^. We found that gracillin showed minimal effects on the mitochondrial function of human lung epithelial cells. Administration of gracillin exhibited no overt side effects with respect to body weight changes, behavioral changes, or functional changes in several organs, including the liver and kidney, and gracillin did not cause several systemic problems, such as metabolic disorders, systemic inflammation, or cellular injuries. Moreover, gracillin is expected to possess low BBB permeability, indicating that gracillin may have a low possibility of causing central nervous system (CNS) or cardiovascular disorders, unlike previously developed CII inhibitors. All of these results clearly suggest a reduced toxic effect of gracillin.

Importantly, gracillin exhibited potent inhibitory effects in cells carrying mutant *KRAS* that is implicated in several types of cancer, including lung, colorectal, endometrial, and pancreatic adenocarcinoma^52^. Despite numerous efforts toward the development of *KRAS*-targeting anticancer drugs for decades, there are no anticancer therapeutics targeting mutant *KRAS* in the clinic^53^; thus, additional strategies targeting *KRAS*-mediated cellular alterations such as metabolic rewiring have been suggested^54^. Indeed, in pancreatic cancer, mutant *KRAS* causes metabolic reprogramming to increased dependence on aerobic glycolysis and glutamine metabolism^54^. In mitochondrial metabolism, decreases in components of CI and mitochondrial energy production have been found in *KRAS*-transformed fibroblasts^55^. Since both CI and CII mediate electron transfer to CIII^31^ and because mitochondrial metabolism and consequent ROS generation are crucial for *KRAS*-induced tumorigenesis^56^, it is possible that cancer cells carrying mutant *KRAS* may acquire alternative strategies such as upregulation of CII to maintain essential mitochondria functions; furthermore, relatively increased dependence on CII activity may be a mechanism underlying the increased sensitivity to gracillin treatment in *KRAS* mutant cancer cells. These findings suggest that gracillin is potentially applicable for the treatment of *KRAS* mutant cancer and that targeting CII may be an alternative strategy for the treatment of *KRAS* mutant cancer. In addition, because gracillin activates AMPK in NSCLC cells expressing mutated *STK11* and potently suppressed the viability of these cells, gracillin can be utilized for the treatment of NSCLC harboring mutant *STK11*. Importantly, recent studies have reported that mutations in *STK11* confer resistance to immune checkpoint inhibitors, especially in *KRAS*-mutant NSCLC^57,58^. Therefore, gracillin can be considered as a potential lead compound to develop adjuvant anticancer drugs for improving anticancer efficacy of clinically available anticancer drugs such as immune checkpoint inhibitors. Additional studies would be necessary to evaluate whether treatment with gracillin in combination can improve the antitumor efficacy of immune checkpoint inhibitors.

Taken together, the results of this study demonstrate the potential of gracillin as an effective anticancer agent targeting CII. Further studies are warranted to evaluate the effectiveness and toxicity of gracillin in additional preclinical and clinical settings.

## Materials and methods

### Chemicals and reagents

Cell culture reagents were purchased from Welgene (Daegu, Republic of Korea). Mouse monoclonal antibodies against cleaved poly (ADP-ribose) polymerase (PARP) were purchased from BD Biosciences (San Jose, CA, USA). Rabbit polyclonal antibodies against pAMPK (T172), AMPK, pmTOR (S2448), mTOR, p70S6K (T389), p70S6K, pS6, S6, and cleaved caspase 3 were purchased from Cell Signaling Technology (Danvers, MA, USA). Primary antibodies against actin and SDHA were purchased from Santa Cruz Biotechnology (Santa Cruz, CA, USA). Rabbit polyclonal SDHB antibody and horseradish peroxidase (HRP)-conjugated secondary antibodies were purchased from GeneTex (Irvine, CA, USA). Primary antibodies against SDHB and SDHC were purchased from Abcam (Cambridge, UK). 3-(4,5-dimethylthiazol-2-yl)-2,5-diphenyl tetrazolium bromide (MTT), propidium iodide (PI), and other chemicals were purchased from Sigma-Aldrich (St. Louis, MO, USA) unless otherwise specified.

### Test compounds

We used a repositioned chemical library consisting of various compounds representative of each chemical class of natural products-derived compounds, including alkaloids, flavonoids, tannins, lignans, and terpenoids. These chemicals are listed in Supplementary Table 1. Large amounts of gracillin were purchased from Chengdu Biopurity Phytochemical Ltd (Sichuan, China).

### Cell culture

Human cancer cell lines were purchased from American Type Culture Collection (ATCC, Manassas, VA, USA) or Korean Cell Line Bank (KCLB) or kindly provided by Dr. John V. Heymach (MD Anderson Cancer Center, Houston, TX, USA). Human bronchial epithelial (HBE) cells were generously provided by Dr. John Minna (The University of Texas Southwestern Medical Center, Dallas, TX). BEAS-2B human HBE cells and 1799 premalignant HBE cells were kindly provided by Dr. A. Klein-Szanto (Fox Chase Cancer Center, Philadelphia, PA). Human cancer cells were cultured in Dulbecco’s Modified Eagle Medium (DMEM) or RPMI 1640 medium supplemented with 10% fetal bovine serum (FBS) and antibiotics. Normal HBE cells were maintained in K-SFM (Invitrogen) supplemented with 5 ng/ml recombinant EGF, 50 mg/mL bovine pituitary extracts, and antibiotics. Cells were maintained at 37 °C with 5% CO_2_ in a humidified atmosphere. Drug-resistant sublines (designated ‘/R’) were generated by exposure of cells to gradually increasing concentrations of chemotherapy (paclitaxel for H226B/R, H460/R, SK-MES-1/R, and DU145/R) or molecular targeted therapy (gefitinib for PC9/GR; erlotinib for PC9/ER) for over six months. Human cancer cell lines were authenticated and validated using AmplFLSTR identifier PCR Amplification Kit (Applied Biosystems, Foster, CA; cat. No. 4322288) in 2013 and 2016. Cells passed for fewer than 3 months after receipt or resuscitation of validated cells were used in this study.

### MTT assay

Cells were seeded into 96-well plates at a density of 2 × 10^3^ cells/well and incubated for 24 h. The cells were then treated with vehicle or test compounds diluted in complete media for three days. Cells were treated with the MTT solution (at a final concentration of 250–500 μg/ml) and incubated for 2 h at 37 °C. The formazan products were dissolved in DMSO, and the absorbance was measured at 570 nm. The data are presented as a percentage of the control group.

### Crystal violet assay

Cancer cells were seeded into 96-well plates at a density of 2–2.5 × 10^3^ cells/well and incubated for 24 h. The cells were then treated with vehicle or test compounds diluted in complete media for three days. After incubation, the cells were fixed with 100% methanol for 30 min at room temperature, and then the plates were dried in air. Fixed cells were stained with 0.01% crystal violet for 30 min at room temperature and then washed with tap water several times. Stained cells were dissolved in 100% methanol, and the absorbance was measured at 570 nm. The data are presented as a percentage of the control group.

### Anchorage-dependent colony formation assay

To determine the effect of test compounds on the anchorage-dependent colony formation, cells were seeded into 6-well plates at a density of 300 cells/well and treated with various concentrations of test compounds diluted in complete medium. The cells were treated for 1–2 weeks, and the medium was changed every three days. At the end of the experiment, cells were fixed with 100% methanol for 15 min, air-dried, and stained with 0.01% crystal violet for 30 min at room temperature. After washing several times with deionized water, colonies were counted using ImageJ software (National Institute of Health, Bethesda, MA, USA)^59^.

### Determination of ATP production

Intracellular ATP levels were determined using ATPlite^TM^ (PerkinElmer, Waltham, MA, USA) according to the manufacturer’s recommended procedure. Cells were seeded into black, clear-bottom 96-well plates (Corning, Corning, NY, USA) at a density of 1000 cells/well and incubated for 24 h. Cells were treated with test compounds for 6 h and then lysed with lysis buffer. Lysates were incubated with the ATPlite substrate, and luminescence was then measured on a SpectraMax M5 multimode microplate reader (Molecular Devices, San Jose, CA, USA).

### Determination of cellular oxidative stress and calcium

Cells were treated with various concentrations (0, 1, 5, and 10 μM) of gracillin for 6 h at 37 °C. 1 h before measurement, the cells were stained with 20 μM H_2_DCF-DA (for oxidative stress) or 1 μM Fluo-4 AM (all from Thermo Fisher Scientific, Waltham, MA, USA). The fluorescence was measured by a SpectraMax M5 multimode microplate reader (Molecular Devices, San Jose, CA, USA) using an excitation wavelength at 485 nm (for H_2_DCF-DA) or 494 nm (for fluo-4 AM) and an emission wavelength at 535 nm (for H_2_DCF-DA) or 516 nm (for fluo-4 AM).

### Cell cycle analysis

Cells were treated with increasing concentrations of gracillin for three days. Adherent and floating cells were collected and fixed in ice-cold 100% methanol overnight at −20 °C. Cells were stained with a propidium iodide (50 μg/ml) solution containing RNase A (50 μg/ml) for 30 min at room temperature. Apoptotic cells were analyzed by flow cytometry using a FACSCalibur® flow cytometer (BD Biosciences).

### Western blot analysis

Cells were harvested with modified RIPA lysis buffer [50 mM Tris-HCl (pH 7.5), 150 mM NaCl, 1 mM EDTA, 0.25% sodium deoxycholate, 1% Triton X-100, 1 mM Na_3_VO_4_, 100 mM NaF, 0.5 mM DTT, 1 mM PMSF, 1 μg/ml aprotinin, 1 μg/ml leupeptin, and 20 mM β-glycerophosphate]. The lysates were centrifuged at 13,000 rpm for 30 min at 4 °C. Equal amounts of protein were separated by 8–10% SDS-PAGE and transferred onto PVDF membranes. Membranes were blocked with blocking buffer (3% BSA in Tris-buffered saline-0.1% Tween-20 (TBST) containing 0.02% sodium azide) for 1 h at room temperature. Membranes were then incubated with a primary antibody (1:1000 dilution in blocking buffer) overnight at 4 °C. The membrane was washed four times with TBST for 1 h at room temperature and then incubated with the corresponding secondary antibody (1:5000 dilution in 3% skim milk in TBST). Membranes were washed four times with TBST for 1 h at room temperature. The blots were visualized using an enhanced chemiluminescence (ECL) detection kit (Thermo Fisher Scientific).

### Fluorescence imaging analysis

Cells were seeded onto black 96-well plates with clear bottom and allowed to attach overnight. The cells were treated with gracillin for 6 h and then stained with the indicated fluorescence dye [100 nM TMRM, 5 μM Mitosox, or 20 μM H_2_DCF-DA, (all from Thermo Fisher Scientific, Waltham, MA, USA)] for 15 (for Mitosox) or 30 min (for others). Cells were counterstained with Hoechst 33342 (1 μg/ml). Fluorescent images were analyzed using the Operetta High Content Imaging System (PerkinElmer).

### Isolation of mitochondria

Isolation of mitochondria was performed as described previously^60^. Confluent H226B and H460 cells were washed twice with PBS and then harvested with PBS by scraping. After centrifugation at 700 × *g* for 5 min at 4 °C, supernatants were removed, and pellets were suspended in MIB buffer [10 mM Tris/MOPS (pH 7.4), 200 mM sucrose, and 1 mM EGTA]. Cells were disrupted by brief sonication and passing through 18-gauge and 27-gauge needles seven times. After centrifugation at 600 × *g* for 5 min at 4 °C, supernatants were collected into new microcentrifuge tubes and further centrifuged at 10,000 × *g* for 5 min at 4 °C. Pellets (mitochondria) were suspended in MIB buffer (for ATP measurement) or MAS3 buffer [3 mM HEPES (pH 7.2–7.4), 10 mM KH_2_PO_4_, 115 mM KCl, 2 mM MgCl_2_, 1 mM EGTA, and 0.2% fatty acid-free bovine serum albumin (BSA); for measurement of the oxygen consumption rate (OCR)] and used for further assays.

### Cloning and extraction of recombinant His-tagged SDHA protein

The human *SDHA* gene was cloned into pET28a vectors with a C-terminal (His)_6_ tag. E. coli BL21 cells were transformed with the construct and were cultured in lysogeny broth (LB) medium at 37 °C until the absorbance at 600 nm reached 0.6. Protein expression was induced by adding 0.3 mM isopropyl-β-d-thiogalactopyranoside (IPTG) and then incubating at 30 °C for 6 h. After centrifugation, cell pellets were resuspended in lysis buffer [50 mM Tris–HCl (pH 8.0), 150 mM NaCl, 1% Triton X-100, 10% glycerol, 70 μl of β-mercaptoethanol, 100 μg/ml lysozyme and protease inhibitor cocktail]. After sonication and centrifugation, the supernatant was incubated with Ni-NTA agarose beads and kept at 4 °C.

### Immunoprecipitation and pull-down assay

For immunoprecipitation analysis, H226 cells treated with vehicle or 5 μM gracillin for 6 h were washed with ice-cold PBS twice and then harvested by IP lysis buffer [40 mM Tris-HCl (pH 7.4), 150 mM NaCl, 0.5% NP-40, 2 mM EDTA, 1 mM MgCl_2_, 5% glycerol, 100 mM NaF, 5 mM Na_3_VO_4_, 1 μg/ml aprotinin, 1 μg/ml leupeptin, and 1 μg/ml pepstatin] for 10 min on ice. After centrifugation at 18,000 × *g* for 10 min at 4 °C, supernatants were harvested, and protein concentration was determined by the BCA assay. One milligramof protein was immunoprecipitated with anti-SDHA antibodies overnight at 4 °C in lysis buffer. Protein G agarose beads were added and incubated for additional 2 h. The beads were collected by centrifugation (1000 × *g* for 5 min at 4 °C) and washed six times (three times with lysis buffer and three times with PBS). Bound proteins were extracted by boiling with 2× SDS-PAGE sample buffer for 5 min at 95 °C. Proteins were resolved by SDS-PAGE, transferred onto PVDF membranes, and then subjected to Western blot analysis as described above.

For pull-down assay, H226B cell lysates were incubated with recombinant His-tagged SDHA protein bound with Ni-NTA agarose beads (Thermo Fisher Scientific) (total volume of 0.5 ml) in the presence of vehicle (DMSO) or 5 μM gracillin in IP lysis buffer for 4 h at 4 °C. Beads were washed 6 times as described above, and proteins were extracted by boiling with 2× SDS-PAGE sample buffer for 5 min at 95 °C. Proteins were analyzed as described above.

### Measurement of mitochondrial respiration

To determine oxygen consumption rates in mitochondria of cancer cells, we used Seahorse XF analyzer (Agilent, Santa Clara, CA, USA) according to the instruction provided by manufacturer.

### LC/MS-based analysis of TCA cycle-related metabolites

H460 and H226B cells (50–60% confluence) were treated with 5 μM gracillin diluted in complete medium for 8 h. Cells were harvested and further analyzed as described previously^61^.

### Succinate dehydrogenase (SDH) activity assay

An SDH activity assay was performed according to the previous literature with modification^33^. Briefly, H460 and H226B cells (1 × 10^4^ cells/well in a 96-well plate) were treated with 500 μg/ml MTT solution in PBS containing 20 mM succinic acid (pH 7.4). Various concentrations of gracillin were added simultaneously and further incubated for 4 h at 37 °C. The medium was removed, and the formazan products were dissolved in DMSO. Absorbance was measured at 570 nm.

### Succinate ubiquinone reductase (SQR) activity assay

The SQR activity assay was performed according to previously reported methods with modifications^62^. Confluent H226B cells in 100 mm culture dishes were washed twice with ice-cold PBS and harvested by scraping in PBS. After centrifugation, cell pellets were suspended in 1 ml of ice-cold 10 mM Tris (pH 7.4) and mechanistically disrupted by sonication using a Vibra-cell ultrasonic processor (Sonics and Materials, Inc., Newtown, CT, USA). After adding 0.2 ml of ice-cold 1.5 M sucrose, the suspension was centrifuged at 600 × *g* for 10 min at 4 °C. Supernatants were further centrifuged at 14,000 × *g* for 10 min at 4 °C. Pellets were suspended in 100 μl of 10 mM Tris (pH 7.4). The protein concentration was determined using a BCA assay kit (Thermo Fisher Scientific). The mitochondria-enriched fraction (20 μl, containing 20 μg of protein) was incubated with 960 μl of the incubation mixture containing 80 mM potassium phosphate (pH 7.8), 1 g/l fatty acid-free BSA, 2 mM EDTA, 0.2 mM ATP, 60 μM 2,6-dichloroindophenol (DCIP), 50 μM decylubiquinone, 1 μM antimycin A, and 3 μM rotenone for 1 min at room temperature. The mixture was treated with vehicle or test compounds (5 μM gracillin or 100 μM 2-thenoyltrifluoroacetone). After measuring the absorbance at 600 nm, 10 μl of 1 M succinate and 10 μl of 30 mM potassium cyanide (KCN) solution (final concentration of 10 mM succinate and 0.3 mM KCN) was added, and the absorbance was monitored at 1 min intervals for 10 min. The enzyme activity of the test group was calculated according to the following formula and expressed as a percentage of the vehicle-treated control group: enzyme activity (nmol/min/mg) = (ΔAbsorbance/min × 1000)/[(extinction coefficient of DCIP × volume of sample used in 1 ml) × (sample protein concentration in mg/ml)]^62^. The extinction coefficient of DCIP was 19.1 mM^−1^cm^−1^.

### Blue native polyacrylamide gel electrophoresis (BN-PAGE) and in-gel enzyme activity assay

The BN-PAGE was performed as described previously with modifications^35,36^. Briefly, isolated mitochondria were resuspended in lysis buffer (50 mM Tris-HCl, pH 7.0, 750 mM aminocapoic acid, and 1.7% *n*-dodecyl-β-*d*-maltoside) and incubated for 10 min on ice. After centrifugation at 10,000 × *g*, 4 °C for 30 min, the protein concentrations were measured using the bicinchoninic acid assay reagents (Thermo Fisher Scientific). The gel loading sample were prepared by adding 10× sample buffer (750 mM aminocaproic acid and 5% Coomassie blue brilliant G-250) to the 10–50 μg of mitochondrial lysate. The BN-PAGE was performed by running samples at 80 V/150 V on a Native PAGE Novex 4–16% gels (Thermo Fisher Scientific) and using separate anode (50 mM Bis–Tris, pH 7.0) and cathode buffer (5 mM Bis–Tris and 50 mM Tricine with or without 0.02% Coomassie blue brilliant G-250). For in-gel enzyme activity assay, light blue cathode buffer (0.002% CBBG) was used. In-gel complex II activity was determined by incubating the BN-PAGE gels in 20 mM Sodium succinate (Sigma), 25 mg Nitrotetrazolium Blue Chloride, 200 μM Phenazoine methosulfate until appearance of violet band. Reaction was stopped with 10% acetic acid. Densitometric analysis to determine the difference in in-gel SDH activity was performed using ImageJ software.

### Animal experiments

All animal experiments were performed according to protocols approved by Seoul National University Institutional Animal Care and Use Committee. Mice were fed standard mouse chow and water ad libitum and housed in temperature-controlled and humidity-controlled facilities with a 12-h light/12-h dark cycle. For xenograft experiments, cells (diluted in equal amount of PBS and Matrigel) or small pieces or tumors were subcutaneously injected into the right flank of 6-week-old female NOD/SCID mice. After the tumor volume reached 50–150 mm^3^ on average, the mice were randomly grouped and orally administered with vehicle (1% DMSO and 9% ethanol in distilled water for cell line xenografts and lung PDX; 5% DMSO in corn oil for colon PDX) or gracillin (10 mg/kg for cell line xenografts and lung PDX; 20 mg/kg for colon PDX) 6 days per week for 2 weeks. Mice failed to develop tumors were not included in the experimental groups. Tumor growth was determined by measuring the short and long diameter of the tumor with a caliper, and body weight was measured twice per week to monitor toxicity. Administration of drugs (vehicle and gracillin) and measurement of tumor growth were performed in a blind fashion. In addition, to evaluate the effect of gracillin on mutant KRAS-driven lung tumorigenesis, two-month-old male and female *Kras*^*G12D/+*^ transgenic mice on the FVB background were randomized and treated with vehicle (1% DMSO and 9% ethanol in distilled water) or gracillin (10 mg/kg) for 8 weeks. The mice were euthanized, and tumor formation was evaluated and compared with that of the vehicle-treated control group as described previously^63^. Briefly, microscopic evaluations of lung tissue were also performed to measure mean tumor number (N) and volume (V) in a blinded fashion after hematoxylin and eosin (H&E) staining. The number and size of tumors were calculated in five sections uniformly distributed throughout each lung. In both animal experiments, the tumor volume was calculated using the following formula: tumor volume (mm^3^) = (short diameter)^2^ × (long diameter) × 0.5.

### Toxicity test

Blood was collected from euthanized *Kras*^*G12D/+*^ transgenic mice treated with vehicle or gracillin under an isoflurane-induced deep anesthesia by cardiac puncture. After allowing blood coagulation at 4 °C, serum was collected by centrifugation at 3000 rpm for 10 min at 4 °C. Analysis of the level of glutamic-oxaloacetic transaminase (GOT), blood urea nitrogen (BUN), creatinine, alanine aminotransferase (GPT), alkaline phosphatase (ALP), glucose, total protein (TP), and albumin (ALB) in the serum was performed using a veterinary hematology analyzer (Fuji DRI-Chem 3500 s, Fujifilm, Tokyo, Japan) according to the manufacturer’s provided protocols. To determine lipid peroxidation in the tissues, the TBARS (thiobarbituric acid reactive substance) assay) assay was performed as follows. Briefly, 200 μl of tissue homogenates or standard [1,1,3,3-tetraethoxypropane (TEP)] solution was mixed with 200 μl of PBS and 200 μl of 0.75% 2-thiobarbituric acid (TBA) in 1N hydrochloric acid solution. After vortexing, the mixture was boiled for 0.5–1 h at 90 °C in thermomixer. After cooling on ice, the absorbance was measured at 540 nm.

### Statistical analysis

The data are presented as the mean ± SD. All experiments were independently performed at least twice, and a representative result is presented. The values presented in the graphs are generated by multiple replicates in a representative experiment. The number of technical replicates in each figure is indicated in figure legends. No statistical methods were used to determine the sample size for in vitro and in vivo experiments. The data were calculated and analyzed using Microsoft Excel software (Microsoft Corp., Redmond, MA, USA) and GraphPad Prism (version 8, GraphPad Software Inc., San Diego, CA, USA). Statistical significance was determined using a two-tailed Student’s *t*-test, one-way ANOVA with Tukey’s multiple comparisons test, Brown-Forsythe and Welch ANOVA tests with Dunnett’s T3 multiple comparisons test, Kruskal-Wallis test with Dunn’s multiple comparisons test, or Mann Whitney test. An F-test for equality of variances was performed to ensure the same variance of two test groups. The Shapiro-Wilk test or D’Agostino and Pearson test was performed to determine whether the data follows a normal distribution. A *P* value of less than 0.05 was considered significant.

## Supplementary information

Supplementary Information No further amendments required.

Supplementary Figure legends No further amendments required

Supplementary Figure 1 No further amendments required

Supplementary Figure 2 The label ‘μM' is missing from the x axis. Please check the attached file (Revised Suppl Fig 2). Thank you.

Supplementary Figure 3 No further amendments required

Supplementary Figure 4 No further amendments required

Contribution form No further amendments required
