# Supplementary material for: The natural compound gracillin exerts potent antitumor activity by targeting mitochondrial complex II
Source: Cell Death Dis. 2019 Oct 24;10(11):810. doi: 10.1038/s41419-019-2041-z (PMC6813327; doi:10.1038/s41419-019-2041-z)
Supplement: Supplementary file 1 — Supplementary Information No further amendments required. [file 41419_2019_2041_MOESM1_ESM.pdf]

Supplementary Information

The natural compound gracillin exerts potent antitumor activity by targeting mitochondrial complex II

Hye-Young Min, Hyun-Ji Jang, Kwan Hee Park, Seung Yeob Hyun, So Jung Park, Ji Hye Kim, Jaekyoung Son, Sam Sik Kang, Ho-Young Lee

Supplementary Table 1. Effects of a natural product chemical library on the viability of NSCLC cells.

No,	Code No.	Class	Name	Cell viability (%)		
				H1299	A549	H460
1	F1.1	Flavonoid	acacetin	124	97.1	86.0
2	F1.2	Flavonoid	amentoflavone	82	94.0	93.4
3	F1.3	Flavonoid	amurenoside B	102	102.1	98.4
4	F1.4	Flavonoid	anhydroicaritin 3 -O- rhamnoside	34	77.9	69.8
5	F1.5	Flavonoid	apigenin	91	107.7	77.4
6	F1.6	Flavonoid	apigenin7-O-glucuroside	89	119.0	102.9
7	F1.7	Flavonoid	astragalin (kaempferol - O - glucoside)	103	101.7	110.3
8	F1.8	Flavonoid	astrapterocarpan 3 - O - glucoside	85	99.6	76.0
9	F1.9	Flavonoid	avicularin (quercetin 3 -O - arabinofuranoside)	100	103.0	96.2
10	F1.10	Flavonoid	baicalin	87	94.2	92.8
11	F1.11	Flavonoid	baicalein	96	59.5	44.9
12	F1.12	Flavonoid	bilobetin	53	100.1	49.8
13	F1.13	Flavonoid	broussochalcone A	64	65.8	52.6
14	F1.14	Flavonoid	calycosin	111	109.4	91.9
15	F1.15	Flavonoid	calycosin 7 - O - glucoside	87	93.8	100.4
16	F1.16	Flavonoid	(+)-catechin	89	94.3	91.1
17	F1.17	Flavonoid	chrysin	120	90.7	74.1
18	F1.18	Flavonoid	clovin	94	98.5	80.4
19	F1.19	Flavonoid	daidzein	86	87.3	71.7
20	F1.20	Flavonoid	daidzin	96	87.8	77.5
21	F1.21	Flavonoid	7,8-dihydroxyflavone	79	82.5	61.3
22	F1.22	Flavonoid	4',7-dihydroxyflavone	104	94.0	89.2
23	F1.23	Flavonoid	diosmetin 7 - O - glucoside	78	84.0	85.9
24	F1.24	Flavonoid	diosmin	110	97.0	95.5
25	F1.25	Flavonoid	echinoisoflavanone	106	71.9	76.6
26	F1.26	Flavonoid	echinoisosophoranone	73	48.1	64.2
27	F1.27	Flavonoid	epicatechin acetate	109	82.9	90.0
28	F1.28	Flavonoid	(-)-epicatechin 3 - O - gallate	122	92.7	96.0
29	F1.29	Flavonoid	epimedin A	101	95.3	89.6
30	F1.30	Flavonoid	epimedin B	91	91.9	95.1
31	F1.31	Flavonoid	epimedin C	114	85.0	93.8
32	F1.32	Flavonoid	epimedoside A	120	85.5	90.1
33	F1.33	Flavonoid	(-)-eriodictyol	117	103.5	85.3
34	F1.34	Flavonoid	eupatilin	94	92.1	71.3
35	F1.35	Flavonoid	evodioside B	116	70.8	68.8
36	F1.36	Flavonoid	formononetin	119	90.0	96.2
37	F1.37	Flavonoid	formononetin acetate	118	84.1	91.4
38	F1.38	Flavonoid	galangin	150	84.6	96.8
39	F1.39	Flavonoid	galangin 3 - O- methyl ether	91	82.1	91.6
40	F1.40	Flavonoid	genistein	82	65.5	57.8
41	F1.41	Flavonoid	genistin	77	73.7	69.5
42	F1.42	Flavonoid	ginkgetin	14	24.1	30.1
43	F1.43	Flavonoid	3-hydroxyflavone	72	76.7	69.1
44	F1.44	Flavonoid	hyperin (hyperoside, quercetin 3 -O- galactopyranoside)	89	79.0	93.2
45	F1.45	Flavonoid	icariin	115	75.0	96.3
46	F1.46	Flavonoid	isoliquiritigenin	90	68.7	90.8
47	F1.47	Flavonoid	isomucronulatol 7 - O - glucoside	71	65.5	84.1
48	F1.48	Flavonoid	isoquercitrin	120	70.3	92.4
49	F1.49	Flavonoid	isorhamnetin	120	69.2	88.0

50	F1.50	Flavonoid	isorhamnetin 3 - O -galactoside	137	73.5	90.1
51	F1.51	Flavonoid	isosophhoranone	70	52.6	100.8
52	F1.52	Flavonoid	isoxanthohumol	61	49.9	56.1
53	F1.53	Flavonoid	jaceosidin	89	63.6	105.2
54	F1.54	Flavonoid	kaemferide	107	72.1	91.6
55	F1.55	Flavonoid	kaempferol	87	68.8	98.1
56	F1.56	Flavonoid	trifolin(kaempferol3-O-galactoside)	108	67.2	95.7
57	F1.57	Flavonoid	kaempferol 7-O-glucoside	96	69.8	102.8
58	F1.58	Flavonoid	kaempferol 3-O-(6''-coumaryl-glucosyl)(1->2) rhamnoside	95	69.7	88.8
59	F1.59	Flavonoid	kaempferol 3-O- glucosyl(1->2)rhamnoside	101	77.4	86.4
60	F1.60	Flavonoid	kaempferol 3-O- 2'', 6''-dirhamnosylglucoside	85	79.5	99.6
61	F1.61	Flavonoid	kaempferol 3-O-rutinoside(nicotiflorin)	103	100.2	92.7
62	F1.62	Flavonoid	kenusanone A	91	81.1	88.2
63	F1.63	Flavonoid	kenusanone C	97	77.7	76.1
64	F1.64	Flavonoid	kuraridin	101	70.3	90.4
65	F1.65	Flavonoid	linarin	147	95.9	89.8
66	F1.66	Flavonoid	liquiritin	112	101.6	93.6
67	F1.67	Flavonoid	liquiritigenin	130	96.2	90.1
68	F1.68	Flavonoid	liquiritigenin acetate	123	92.5	117.2
69	F1.69	Flavonoid	luteolin	138	89.1	92.4
70	F1.70	Flavonoid	luteolin 5 - O - glucoside	132	92.1	82.8
71	F1.71	Flavonoid	luteolin 7 - O - glucoside	145	86.0	63.6
72	F1.72	Flavonoid	maackiain	143	77.2	73.6
73	F1.73	Flavonoid	morin	108	93.4	81.5
74	F1.74	Flavonoid	myricetin	100	93.0	86.4
75	F1.75	Flavonoid	naringenin	101	91.5	84.2
76	F1.76	Flavonoid	naringin	106	92.6	91.7
77	F1.77	Flavonoid	neohesperidin	117	92.0	86.6
78	F1.78	Flavonoid	nepetin	69	63.2	51.7
79	F1.79	Flavonoid	ochnaflavone	52	36.9	31.8
80	F1.80	Flavonoid	oroxylin A	91	81.2	75.1
81	F1.81	Flavonoid	pectolinarin	94	80.2	80.8
82	F1.82	Flavonoid	poncirin	125	87.8	91.7
83	F1.83	Flavonoid	puerarin	98	87.5	84.0
84	F1.84	Flavonoid	psoralidin	55	44.9	66.1
85	F1.85	Flavonoid	quercetin	102	89.5	88.9
86	F1.86	Flavonoid	quercitirin	93	82.6	88.0
87	F1.87	Flavonoid	quercetin3-O-(6''-coumaroyl-glucosyl)(1->2)rhamnoside	107	75.4	79.9
88	F1.88	Flavonoid	quercetin3-O-2'',6''-dirhamnosylglucoside	130	89.6	87.5
89	F1.89	Flavonoid	rhoifolin	143	80.3	84.6
90	F1.90	Flavonoid	robinin	86	84.6	82.2
91	F1.91	Flavonoid	rutin	97	84.4	91.8
92	F1.92	Flavonoid	rutin2''-gallate	106	93.2	102.2
93	F1.93	Flavonoid	sciadopitysin	106	81.8	84.5
94	F1.94	Flavonoid	sophoraflavanone D	107	102.9	96.2
95	F1.95	Flavonoid	sophoraflavanone G	72	50.0	56.4
96	F1.96	Flavonoid	sophoraflavescenol	102	53.2	38.4
97	F1.97	Flavonoid	spinosin	109	95.3	109.5
98	F1.98	Flavonoid	6'''-feruloylspinosin	106	99.0	99.1
99	F1.99	Flavonoid	swertisin acetate	112	78.8	98.2
100	F1.100	Flavonoid	2'''-O-syringylrutin	112	93.0	108.0
101	F1.101	Flavonoid	tiliroside	92	81.7	96.7
102	F1.102	Flavonoid	trifolirhizin	97	93.2	86.4
103	F1.103	Flavonoid	vitexicarpin	90	50.3	76.3

104	F1.104	Flavonoid	vitexin	84	101.4	111.7
105	F1.105	Flavonoid	vitexin permethyl ether	103	91.0	92.9
106	F1.106	Flavonoid	wogonin	64	99.5	91.4
107	I2.1	Iridoid	ajunol	124	89.0	100.9
108	I2.2	Iridoid	aucubin	146	93.9	91.8
109	I2.3	Iridoid	catalpol	99	91.9	102.3
110	I2.4	Iridoid	dimethylsecologanoside	113	85.1	100.3
111	I2.5	Iridoid	epivogeloside	90	95.6	126.5
112	I2.6	Iridoid	campsiside	78	82.2	108.6
113	I2.7	Iridoid	harpagide	102	91.0	103.5
114	I2.8	Iridoid	geniposide	146	89.9	108.5
115	I2.9	Iridoid	loganic acid	141	95.3	113.4
116	I2.10	Iridoid	loganin	99	101.0	109.4
117	I2.11	Iridoid	monotropein	123	94.2	112.1
118	I2.12	Iridoid	secologanin dimethyl acetal	143	92.6	89.2
119	I2.13	Iridoid	sweroside	129	104.9	109.9
120	I2.14	Iridoid	aeginetoyl ajugol5''-O- b - D - quinovoside	142	101.0	98.5
121	I2.15	Iridoid	6-O-(4''-O-I-a-L-rhamnosyl)vanilloylajugol	172	95.1	95.6
122	I2.16	Iridoid	valeroside	69	107.4	93.5
123	M3.1	Terpenoid	albiflorin	114	102.9	102.5
124	M3.2	Terpenoid	benzoylpaeoniflorin	112	103.6	94.9
125	M3.3	Terpenoid	galloylpaeoniflorin	114	92.2	100.2
126	M3.4	Terpenoid	lactiflorin	96	98.0	107.8
127	M3.5	Terpenoid	oxypaeoniflorin	121	97.5	91.8
128	M3.6	Terpenoid	paeoniflorin	106	88.9	90.9
129	M3.7	Terpenoid	paeoniflorin acetate	100	90.5	101.5
130	M3.8	Terpenoid	1-O-beta-D-glucopyranosyl-8-O-benzoylpaeonisuffrone	142	99.8	87.1
131	M3.9	Terpenoid	rengyol	140	115.1	94.2
132	S4.1	Terpenoid	artemisinin	89	94.5	80.7
133	S4.2	Terpenoid	bilobalide	157	86.5	98.9
134	S4.3	Terpenoid	handelin	31	79.6	93.6
135	S4.4	Terpenoid	9-hydroxyheterogorgiolide	154	82.8	84.5
136	S4.5	Terpenoid	(+)-ledol	89	80.5	83.8
137	S4.6	Terpenoid	patulialcohol	78	73.9	89.0
138	S4.7	Terpenoid	aeginetic acid 5 - O - beta - D - quinovoside	86	81.3	88.4
139	D5.1	Terpenoid	abietic acid	66	85.0	95.6
140	D5.2	Terpenoid	crocin	75	77.7	93.5
141	D5.3	Terpenoid	tanshinone I	82	110.6	93.2
142	D5.4	Terpenoid	tanshinone IIA	37	65.0	86.6
143	D5.5	Terpenoid	ginkgolide A	85	76.8	94.6
144	D5.6	Terpenoid	ginkgolide B	129	73.9	99.5
145	D5.7	Terpenoid	ginkgolide C	105	75.0	100.0
146	D5.8	Terpenoid	ent-kaur-16-en-19-oic acid (kaurenoic acid)	129	80.7	95.7
147	D5.9	Terpenoid	taxol	61	48.3	62.5
148	D5.10	Terpenoid	taxinine	131	84.2	97.2
149	D5.11	Terpenoid	taxinine A	141	87.4	94.2
150	D5.12	Terpenoid	taxinine B	126	96.7	89.2
151	T6.1	Terpenoid	acacigenin B	92	47.3	70.4
152	T6.2	Terpenoid	acacigenin B methyl ester monoacetate	105	83.1	73.0
153	T6.3	Terpenoid	aleuritolic acid	110	79.2	73.5
154	T6.4	Terpenoid	aleuritolic acid methyl ester	115	104.2	106.9

155	T6.5	Terpenoid	aleuritolic acid methyl acetate	104	88.4	83.8
156	T6.6	Terpenoid	aleuritolic acid acetate	121	77.1	95.5
157	T6.7	Terpenoid	epialeuritolic acid	97	74.5	94.3
158	T6.8	Terpenoid	epialeuritolic acid methyl acetate	128	85.6	99.6
159	T6.9	Terpenoid	beta-amyrin	124	78.5	93.4
160	T6.10	Terpenoid	beta-amyrin acetate	118	80.5	95.9
161	T6.11	Terpenoid	asiatic acid	125	91.8	95.5
162	T6.12	Terpenoid	betulin	128	94.9	115.9
163	T6.13	Terpenoid	betulinic acid	99	62.9	75.0
164	T6.14	Terpenoid	betulinic acid methyl ester	94	72.8	87.6
165	T6.15	Terpenoid	corosolic acid	98	73.0	96.1
166	T6.16	Terpenoid	echinocystic acid	176	74.7	84.4
167	T6.17	Terpenoid	erythrodiol	150	78.7	86.8
168	T6.18	Terpenoid	esculentic acid dimethyl ester	114	67.8	62.2
169	T6.19	Terpenoid	esculentic acid	190	87.1	83.5
170	T6.20	Terpenoid	friedelin	198	91.5	89.0
171	T6.21	Terpenoid	hederagenin acetate	181	72.2	81.2
172	T6.22	Terpenoid	glabrolide	163	74.4	97.5
173	T6.23	Terpenoid	glycyrrhetic acid	138	73.0	73.0
174	T6.24	Terpenoid	glycyrrhetic acid methyl acetate	136	71.3	85.8
175	T6.25	Terpenoid	jaligonic acid	150	76.8	83.9
176	T6.26	Terpenoid	jaligonic acid dimethyl ester	105	54.2	59.6
177	T6.27	Terpenoid	jaligonic acid 28-monomethyl ester	170	79.4	83.1
178	T6.28	Terpenoid	liquiritic acid	160	90.3	86.7
179	T6.29	Terpenoid	lupeol	181	73.0	110.1
180	T6.30	Terpenoid	lupenone	201	75.7	114.4
181	T6.31	Terpenoid	mesembryanthemoidigenic acid	87	78.8	106.3
182	T6.32	Terpenoid	myricadiol 3 - acetate	130	72.2	112.3
183	T6.33	Terpenoid	30-norarjunolic acid	112	76.2	104.4
184	T6.34	Terpenoid	30-norhederagenin	139	76.7	104.5
185	T6.35	Terpenoid	oleanolic acid	89	75.7	87.6
186	T6.36	Terpenoid	oleanolic acid methyl ester	79	65.3	97.5
187	T6.37	Terpenoid	oleanolic acid acetate	82	71.2	116.3
188	T6.38	Terpenoid	oleanolic acid methyl acetate	100	71.6	115.7
189	T6.39	Terpenoid	oleanolic acid 3 - keto	134	48.6	82.7
190	T6.40	Terpenoid	panaxadiol	159	72.0	102.0
191	T6.41	Terpenoid	panaxatriol	139	76.3	101.1
192	T6.42	Terpenoid	beta-peltoboykinolic acid methyl acetate	167	74.4	120.0
193	T6.43	Terpenoid	phytolaccagenic acid	143	70.5	108.9
194	T6.44	Terpenoid	phytolaccagenin	193	67.0	91.4
195	T6.45	Terpenoid	phytolaccagenin triacetate	181	56.8	84.6
196	T6.46	Terpenoid	soyasapogenol B triacetate	142	67.9	90.2
197	T6.47	Terpenoid	tormentic acid	209	77.7	94.3
198	T6.48	Terpenoid	ursolic acid	96	48.3	74.1
199	T6.49	Terpenoid	3 alpha - hydroxyoleanolic acid methyl ester	79	78.6	99.3
200	T6.50	Terpenoid	3 beta, 21beta, 30- trihydroxyolean-12-en	77	74.9	96.0
201	T6.51	Terpenoid	taraxerol	87	70.4	95.1
202	T6.52	Terpenoid	betulafoliane diol	73	89.8	99.0
203	T6.53	Terpenoid	23-dihydroganoderic acid I	90	85.1	98.5
204	T6.54	Terpenoid	24-dihydroganoderic acid N	79	89.2	92.5
205	T6.55	Terpenoid	limonin	96	86.5	96.5
206	T6.56	Terpenoid	obacunone	79	79.7	84.3

207	T6.57	Terpenoid	lanosterol	77	86.3	88.0
208	T6.58	Terpenoid	pomolic acid 3 - acetate	118	74.5	89.2
209	T6.59	Terpenoid	fraxinellone	108	90.8	92.4
210	T6.60	Terpenoid	pomonic acid	102	77.6	86.1
211	T6.61	Terpenoid	isotetrahydrofraxinellone	157	93.0	86.3
212	T6.62	Terpenoid	hexahydrofraxinellone	111	91.4	87.8
213	S7.1	Terpenoid	diosgenin	73	92.0	74.7
214	S7.2	Terpenoid	diosgenin acetate	70	89.1	80.2
215	S7.3	Terpenoid	gitogenin	72	75.2	69.1
216	S7.4	Terpenoid	hecogenin	88	83.5	77.8
217	S7.5	Terpenoid	hecogenin acetate	107	88.9	85.1
218	S7.6	Terpenoid	(25S)-ruscogenin	111	115.2	102.7
219	S7.7	Terpenoid	sarsasapogenin	96	85.4	73.4
220	T8.1	Terpenoid	astragaloside I	82	83.4	86.5
221	T8.2	Terpenoid	astragaloside II	78	95.2	96.7
222	T8.3	Terpenoid	astragaloside II	99	85.7	87.4
223	T8.4	Terpenoid	astragaloside IV	90	79.7	83.2
224	T8.5	Terpenoid	azukisaponin II	141	86.5	76.3
225	T8.6	Terpenoid	azukisaponin V	92	83.6	80.4
226	T8.7	Terpenoid	azukisaponin V methyl ester	86	86.5	84.9
227	T8.8	Terpenoid	chikusetsusaponin IV	99	88.2	90.1
228	T8.9	Terpenoid	ginsenoside Rg1	133	90.4	96.3
229	T8.10	Terpenoid	jujuboside A2	74	86.0	87.4
230	T8.11	Terpenoid	loniceroside A	78	82.6	92.4
231	T8.12	Terpenoid	narcissiflorine 6'-methyl ester	124	85.0	87.6
232	T8.13	Terpenoid	narcissiflorine dimethyl ester	113	77.5	103.7
233	T8.14	Terpenoid	niga-ichigoside F1	93	87.2	82.6
234	T8.15	Terpenoid	oleanolic acid 3-O-glucuronoside	112	87.4	94.7
235	T8.16	Terpenoid	oleanolic acid 3-O-glucuroside dimethyl ester	97	64.2	58.6
236	T8.17	Terpenoid	oleanolic acid 28-O-glucuronoside	78	87.1	102.6
237	T8.18	Terpenoid	phytolaccoside B	146	88.2	97.6
238	T8.19	Terpenoid	phytolaccoside E	115	88.0	97.3
239	T8.20	Terpenoid	phytolaccoside F	140	95.4	98.3
240	T8.21	Terpenoid	phytolaccoside I	111	96.5	96.2
241	T8.22	Terpenoid	platycodin D	103	33.2	100.3
242	T8.23	Terpenoid	platycoside E	130	99.7	104.0
243	T8.24	Terpenoid	pulsatilla saponin F	123	95.9	106.7
244	T8.25	Terpenoid	pulsatilla saponin H	121	116.6	104.1
245	T8.26	Terpenoid	salsoloside C methyl ester	134	82.1	91.8
246	T8.27	Terpenoid	silphioside A	126	84.1	100.1
247	T8.28	Terpenoid	soyasaponin II methyl ester	136	102.5	97.1
248	T8.29	Terpenoid	stipleanoside R1 dimethyl ester	162	98.2	105.2
249	T8.30	Terpenoid	stipleanoside R2	130	85.6	97.0
250	T8.31	Terpenoid	stipleanoside R2 methyl ester	149	92.5	92.2
251	T8.32	Terpenoid	suavissimoside R1	115	88.5	100.3
252	T8.33	Terpenoid	ziyuglycoside I	112	82.4	96.5
253	T8.34	Terpenoid	ziyuglycoside II	92	79.5	88.4
254	S9.1	Terpenoid	anemarsaponin B	92	95.0	94.3
255	S9.2	Terpenoid	diosgegnin 3-O-rhamnosyl(1->2) glucoside	16	21.6	16.4
256	S9.3	Terpenoid	dioscin	13	21.5	17.1
257	S9.4	Terpenoid	gracillin	14	20.9	21.4
258	S9.5	Terpenoid	fenugreek saponin II	88	105.6	103.0
259	S9.6	Terpenoid	spicatoside A	119	128.1	112.8

260	S9.7	Terpenoid	trillin	83	75.5	74.4
261	L10.1	Lignan	americanin A	50	79.8	88.4
262	L10.2	Lignan	acetonylidene americanin A	71	78.3	77.4
263	L10.3	Lignan	americanin B	78	82.6	76.1
264	L10.4	Lignan	dimethyl lithospermic acid	90	96.7	97.0
265	L10.5	Lignan	gomisin A	105	87.2	99.3
266	L10.6	Lignan	gomisin N	104	83.0	94.6
267	L10.7	Lignan	honokiol	94	75.2	93.6
268	L10.8	Lignan	matairesinoside	89	88.7	78.2
269	L10.9	Lignan	paulownin	112	100.7	76.6
270	L10.10	Lignan	pinoresicol glucoside	102	95.6	77.9
271	L10.11	Lignan	schizandrin	119	101.7	90.8
272	L10.12	Lignan	schisantherin A (gomisin C)	86	114.0	76.8
273	L10.13	Lignan	schisantherin C	197	90.5	66.8
274	L10.14	Lignan	sesamin	172	101.3	98.3
275	L10.15	Lignan	sesamol	170	104.3	104.6
276	L10.16	Lignan	sesamolin	78	94.5	81.3
277	L10.17	Lignan	sesangolin	81	99.8	91.2
278	L10.18	Lignan	silybin	172	94.6	77.5
279	L10.19	Lignan	simplexoside	96	96.5	86.1
280	L10.20	Lignan	magnolol	276	130.8	107.8
281	C11.1	Coumarin	angelican	122	114.0	110.1
282	C11.2	Coumarin	bergapten	113	105.1	103.4
283	C11.3	Coumarin	bergaptol acetate	86	88.4	84.5
284	C11.4	Coumarin	coumarin	107	97.6	84.0
285	C11.5	Coumarin	6-methyl coumarin	111	83.4	77.3
286	C11.6	Coumarin	decursin	96	86.8	88.2
287	C11.7	Coumarin	decursinol	99	89.6	88.7
288	C11.8	Coumarin	esculetin	99	98.2	94.1
289	C11.9	Coumarin	esculin	132	99.3	99.7
290	C11.10	Coumarin	glabralactone	70	80.8	71.4
291	C11.11	Coumarin	imperatorin	93	104.1	87.7
292	C11.12	Coumarin	isoimperatorin	79	102.7	88.8
293	C11.13	Coumarin	isooxypeucedanin	92	93.8	88.8
294	C11.14	Coumarin	trans-khellactone	84	98.0	92.4
295	C11.15	Coumarin	nodakenetin	116	88.1	110.5
296	C11.16	Coumarin	modakenin	101	96.3	108.7
297	C11.17	Coumarin	osthol	100	114.1	111.7
298	C11.18	Coumarin	oxypeucedanin	83	101.4	77.3
299	C11.19	Coumarin	oxypeucedanin hydrate	102	114.3	79.4
300	C11.20	Coumarin	oxypeucedanin methanolate	89	106.8	74.1
301	C11.21	Coumarin	pabulenol	100	112.9	75.2
302	C11.22	Coumarin	prangolarin[(+)-oxypeucedanin]	82	121.8	76.3
303	C11.23	Coumarin	psoralen	120	131.9	84.4
304	C11.24	Coumarin	scopletin	105	131.9	84.4
305	C11.25	Coumarin	scopolin	107	122.4	98.1
306	C11.26	Coumarin	umbelliferone	110	100.6	90.2
307	C11.27	Coumarin	xanthotoxin(8-methoxypsoralen)	108	110.2	108.9
308	S12.1	Stilbenoid	trans - resveratrol	107	102.7	71.0
309	A13.1	Alkaloid	N-acetylanonaine[(-)-acetylanonaine]	124	97.3	79.1
310	A13.2	Alkaloid	N-acetylanthranilic acid methyl ester	137	117.4	86.2
311	A13.3	Alkaloid	aconitine	155	129.3	84.8
312	A13.4	Alkaloid	adenosine	117	130.2	102.0
313	A13.5	Alkaloid	ajmalicine	112	80.6	71.6

314	A13.6	Alkaloid	allantoin	176	103.1	91.3
315	A13.7	Alkaloid	amygdaline	122	105.4	85.6
316	A13.8	Alkaloid	berberine-HCl	99	65.7	70.0
317	A13.9	Alkaloid	L--citrulline	159	97.5	91.1
318	A13.10	Alkaloid	confusameline	95	106.1	91.7
319	A13.11	Alkaloid	crassicauline A	87	107.7	84.9
320	A13.12	Alkaloid	dehydroevodiamine-HCl	100	95.3	102.1
321	A13.13	Alkaloid	3-deoxyhokbusine A	111	96.3	102.5
322	A13.14	Alkaloid	dictamine	119	106.1	102.7
323	A13.15	Alkaloid	evodiamine	53	52.6	82.3
324	A13.16	Alkaloid	evolitrine	109	99.5	91.9
325	A13.17	Alkaloid	gamma-fagarine	112	101.7	101.3
326	A13.18	Alkaloid	harmaline(dihydroharmine)	99	110.7	92.4
327	A13.19	Alkaloid	6-hydroxykynurenic acid	109	120.8	114.9
328	A13.20	Alkaloid	hypaconitine	110	89.1	75.6
329	A13.21	Alkaloid	indole 3 -butyric acid	110	104.2	105.9
330	A13.22	Alkaloid	kokusaginine	91	90.3	95.1
331	A13.23	Alkaloid	lycoctonine	110	105.9	95.8
332	A13.24	Alkaloid	magnoflorine	106	107.0	79.8
333	A13.25	Alkaloid	matrine	112	120.3	91.9
334	A13.26	Alkaloid	neoline	133	123.2	96.6
335	A13.27	Alkaloid	nicotinamide	107	112.9	79.1
336	A13.28	Alkaloid	nicotinic acid	108	87.0	90.6
337	A13.29	Alkaloid	oxymatrine	121	99.0	100.6
338	A13.30	Alkaloid	palmatine	97	84.6	84.4
339	A13.31	Alkaloid	piperine	116	100.8	73.4
340	A13.32	Alkaloid	prunasine	112	110.5	89.2
341	A13.33	Alkaloid	ricinine	113	117.9	97.0
342	A13.34	Alkaloid	N-demethylricinine	124	105.2	68.6
343	A13.35	Alkaloid	robustine	129	77.6	91.2
344	A13.36	Alkaloid	rutaecarpine	67	69.0	61.3
345	A13.37	Alkaloid	skimmianine	89	95.4	72.6
346	A13.38	Alkaloid	sophocarpine	110	99.7	95.0
347	A13.39	Alkaloid	synephrine	102	86.9	86.8
348	A13.40	Alkaloid	uridine	98	102.5	95.9
349	P14.1	Phenolic	acteoside	75	99.2	102.3
350	P14.2	Phenolic	androsin	100	91.3	89.1
351	P14.3	Phenolic	arbutin	116	81.0	84.9
352	P14.4	Phenolic	angeliticin A	85	86.7	92.8
353	P14.5	Phenolic	benzoic acid	81	86.0	107.6
354	P14.6	Phenolic	caffeic acid(3,4-dihydroxycinnamin acid)	71	86.9	84.8
355	P14.7	Phenolic	caffeic acid methyl ester	100	92.9	91.1
356	P14.8	Phenolic	caffeic aldehyde	91	100.7	74.3
357	P14.9	Phenolic	chlorogenic acid	72	90.8	101.2
358	P14.10	Phenolic	coniferin	74	105.0	71.5
359	P14.11	Phenolic	corilagin	55	123.4	81.8
360	P14.12	Phenolic	3,5-dicaffeoylquinic acid methyl ester	75	110.7	87.5
361	P14.13	Phenolic	2,5-dihydroxyacetophenone	73	128.7	73.2
362	P14.14	Phenolic	gentisic acid	101	122.8	77.4
363	P14.15	Phenolic	emodin	103	138.1	83.3
364	P14.16	Phenolic	emodin8-O-glucoside	75	147.9	91.0
365	P14.17	Phenolic	ferulic acid	104	134.7	97.8
366	P14.18	Phenolic	gallic acid	86	98.2	76.9
367	P14.19	Phenolic	gallic acid methyl ester	86	107.6	68.7

368	P14.20	Phenolic	gallic aldehyde	97	105.7	62.3
369	P14.21	Phenolic	1-O-galloyl beta-D-glucose	83	136.2	82.2
370	P14.22	Phenolic	gastrodin	111	133.3	95.5
371	P14.23	Phenolic	geraniin	82	131.2	96.2
372	P14.24	Phenolic	hexadecanoylferulate	96	129.6	89.7
373	P14.25	Phenolic	homoarbutin	125	94.0	85.4
374	P14.26	Phenolic	p-hydroxybenzoic acid	84	109.0	76.9
375	P14.27	Phenolic	4-hydroxybenzylmethyl ether	97	109.5	73.2
376	P14.28	Phenolic	hydroxyprenylhomoarbutin	131	111.9	80.2
377	P14.29	Phenolic	isoferulic acid	97	108.6	93.2
378	P14.30	Phenolic	khellin	131	122.0	99.7
379	P14.31	Phenolic	lawsone	190	289.7	168.2
380	P14.32	Phenolic	mangiferin	103	134.7	101.5
381	P14.33	Phenolic	paeonol	97	106.3	100.9
382	P14.34	Phenolic	paeonoside	116	115.0	86.0
383	P14.35	Phenolic	pentagalloyl beta-D-glucose	83	72.9	54.6
384	P14.36	Phenolic	piperic acid methyl ester	131	111.8	88.8
385	P14.37	Phenolic	pirolatin	109	121.4	99.6
386	P14.38	Phenolic	protocatechualdehyde(3,4-dihydroxybenzaldehyde)	103	125.1	87.2
387	P14.39	Phenolic	protocatechuic acid(3,4-dihydroxybenzoic acid)	96	135.0	104.7
388	P14.40	Phenolic	salicin	105	102.3	87.3
389	P14.41	Phenolic	salicyclic acid	94	88.5	75.2
390	P14.42	Phenolic	shikimic acid	77	110.0	82.9
391	P14.43	Phenolic	shikonin	27	63.2	65.2
392	P14.44	Phenolic	sinapic acid	93	86.3	82.8
393	P14.45	Phenolic	syringin	81	82.8	133.0
394	P14.46	Phenolic	syringic acid(4-hydroxy-3,5-dimethoxybenzoic acid)	78	84.5	87.7
395	P14.47	Phenolic	thymol	87	90.5	103.0
396	P14.48	Phenolic	vanillic acid(3-methoxy-4-hydroxybenzoic acid)	80	120.2	80.8
397	P14.49	Phenolic	4-hydroxy-3-methoxycinnamaldehyde	75	115.5	101.0
398	P14.50	Phenolic	vanillin(4-hydroxy-3-methoxybenzaldehyde)	85	107.8	109.6
399	P14.51	Phenolic	2,4-dihydroxybenzoic acid	87	112.2	97.0
400	P14.52	Phenolic	3,4-methoxybenzoic acid	87	108.2	106.6
401	P14.53	Phenolic	3,5-dihydroxybenzoic acid	73	107.4	102.6
402	P14.54	Phenolic	cis-4-hydroxycinnamic acid	90	121.9	111.0
403	P14.55	Phenolic	1,4-dihydroxy-2-naphthoic acid	79	80.6	79.8
404	S15.1	Steroid	cyasterone	107	90.3	101.1
405	S15.2	Steroid	ecdysterone	102	110.1	111.4
406	S15.3	Steroid	ergosterol	77	104.6	112.5
407	S15.4	Steroid	5-dihydroergosterol	82	100.6	90.2
408	S15.5	Steroid	fucosterol	105	135.3	113.7
409	S15.6	Steroid	beta-sitosterol	82	111.4	96.3
410	S15.7	Steroid	beta-sitosterol3-O-glucoside	105	120.5	90.3
411	S15.8	Steroid	6'-palmitoyl-beta-sitosterol3-O-glucoside	78	106.0	111.9
412	S15.9	Steroid	alpha-spinasterol	78	86.1	90.8
413	S15.10	Steroid	17- hydroxy-12-O-benzoyllineolon	78	93.9	85.0
414	S15.11	Steroid	12-O-benzoyllineolon	75	100.0	92.1
415	C16.1	Ceramide, Cerebroside	aralia cerebroside	80	106.0	87.7
416	C16.2	Ceramide, Cerebroside	aralia ceramide	78	110.1	93.2

417	C16.3	Ceramide, Cerebroside	pokeseed ccerebroside	94	110.4	118.0
418	C16.4	Ceramide, Cerebroside	soya-cerebroside I	69	124.2	109.6
419	C16.5	Ceramide, Cerebroside	jio-cerebroside(soyacerebroside I & II)	75	125.8	117.1
420	O17.1	Others	azelaic acid	116	102.6	101.3
421	O17.2	Others	5-(alpha-D-galactopyranosyloxymethl)-2-furancarboxaldehyde	108	100.8	91.5
422	O17.3	Others	1-monopalmitoyl-rac-glycerol(monopalmitin)	110	108.1	89.5
423	O17.4	Others	D-mannitol	96	99.5	95.1
424	O17.5	Others	eleutheroside C (ethyl alpha-D-galactopyranoside)	98	105.8	101.2
425	O17.6	Others	(1R,3R,4R,5R)-(-)-quinic acid	94	120.3	112.4
426	O17.7	Others	tiglic acid	110	109.7	111.5

Supplementary Table 2. The IC_{50} values of gracillin and genetic alterations in each cell line.

Type	Histology[1]	Name	IC_{50} (μM)[2]	Genetic alterations[3]						
				EGFR	*KRAS*	*TP53*	*STK11*	*BRAF*	*PTEN*	*PIK3CA*
Lung		1198	4.03	Un	WT[4]	WT[4]	Un	Un	Un	Un
Lung	ADC	A427	4.77	WT	Mut	WT	Del[5]	Mut[6]	WT	WT
Lung	ADC	H1395	2.17	WT	WT	WT	Del	Mut	WT	WT
Lung	ADC	H1944	4.59	WT	Mut	WT	Mut	WT	WT	WT
Lung	ADC	H1975	1.96	Mut	WT	Mut	WT	WT	WT	Mut
Lung	ADC	H2122	1.84	WT	Mut	Mut	Del	WT	WT	WT
Lung	ADC	H2126	2.32	WT	WT	Mut	Del	WT	WT	WT
Lung	ADC	H292	1.88	WT	WT[7]	WT	WT	WT	WT	WT
Lung	ADC	H322	2.07	WT	WT	Mut	Del	WT	WT	WT
Lung	ADC	HCC827	3.97	Mut	WT	Mut	WT	WT	WT	WT
Lung	ADC	PC9	1.98	Mut	WT	Mut	WT	WT	WT	WT
Lung	ADSQC	A549	2.31	WT	Mut	WT	Mut	WT	WT	WT
Lung	ADSQC	H596	7.5	WT	WT	Mut	WT	WT	WT	Mut
Lung	SQCC	H226	3.62	WT	WT	WT	WT	WT	WT	WT
Lung	SQCC	H226B	4.73	WT	WT	WT	Un	Un	WT	Un
Lung	SQCC	H226Br	5.8	WT	Mut	Mut	Un	Un	Un	Un
Lung	SQCC	HCC15	2.26	WT	WT	Mut	Del	WT	WT	WT
Lung	SQCC	Calu1	2.41	WT	Mut	Null	WT	WT	WT	WT
Lung	SQCC	SK-MES-1	4.59	Mut[8]	WT	Mut	WT	WT	WT	WT
Lung	LCC	H1299	5.35	WT	WT	Null	WT	WT	Null[9]	WT
Lung	LCC	H460	1.84	WT	Mut	WT	Mut	WT	WT	Mut
Lung	LCC	H661	5.5	WT	WT	Mut	WT	Mut[7]	WT	WT
Colorectal		HCT116	2.01	WT	Mut	WT	WT	Mut	WT	Mut
Colorectal		HCT15	2.67	WT	Mut	Mut	WT	WT	WT	Mut
Colorectal		HT29	1.98	WT	WT	Mut	WT	Mut	WT	Mut
Colorectal		LS174T	4.71	WT	Mut	WT	WT	WT	WT	Mut
Colorectal		RKO	2.8	WT	WT	WT	WT	Mut	WT	Mut
Prostate		DU145	3.29	Mut[10]	WT	Mut	Mut	Mut	WT	WT
Prostate		LNCaP	3.92	WT	WT	Mut	WT	WT	Del	WT
Prostate		PC3	2.94	WT	WT	Del	WT	WT	Del	WT
Hypopharynx		FaDu	1.89	WT	WT	Mut	WT	WT	WT	WT
Liver		Huh7	2.36	WT	WT	Mut	WT	WT	WT	WT
Liver		Hep3B	4.22	Un	Un	Mut	Un	Un	Un	Un
Lung		H226B/R	2.16	ND	ND	ND	ND	ND	ND	ND
Lung		H460/R	3.94	ND	ND	ND	ND	ND	ND	ND
Lung		SK-MES-1/R	1.96	ND	ND	ND	ND	ND	ND	ND
Prostate		DU145/R	5.55	ND	ND	ND	ND	ND	ND	ND
Lung		PC9/GR	2.81	ND	ND	ND	ND	ND	ND	ND
Lung		PC9/ER	2.58	ND	ND	ND	ND	ND	ND	ND

[1]ADC: Adenocarcinoma; ADSQC: Adenosquamous carcinoma; SQCC: Squamous cell carcinoma; LCC: large cell carcinoma
[2]The IC_{50} values were determined by non-linear regression analysis using GraphPad Prism 7.
[3]Genetic alterations were retrieved from the Catalogue of Somatic Mutations in Cancer (COSMIC) database, the Cancer Cell Line Encyclopedia (CCLE) database, and literatures. WT: wild type; Mut: mutated; Un: unknown; Del: deletion; ND: not determined.

[4]Mutation status of *KRAS* and *TP53* in 1198 cells was reported in the previous literature[1]
[5]Deletion mutation in *STK11* was reported in the previous literature[2].
[6]*BRAF* mutation in A427 and H661 cells was retrieved from the COSMIC database.
[7]KRAS G12S mutation has been identified in a previous study, but this mutation might be an artifact caused by a long-term culture and should be further validated[3].
[8]*EGFR* mutation in SK-MES-1 cells was retrieved from the COSMIC database.
[9]Undetectable expression caused by promoter methylation[4]
[10]*EGFR* mutation in DU145 cells was retrieved from the COSMIC and the CCLE databases.

References

1. Lacroix, L., Feng, G. & Lotan, R. Identification of genes expressed differentially in an in vitro human lung carcinogenesis model. *Cancer Biol Ther* **5**, 665-673 (2006).

2. Carretero, J., Medina, P.P., Pio, R., Montuenga, L.M. & Sanchez-Cespedes, M. Novel and natural knockout lung cancer cell lines for the LKB1/STK11 tumor suppressor gene. *Oncogene* **23**, 4037-4040 (2004).

3. Deschoolmeester, V.*, et al.* KRAS mutation detection and prognostic potential in sporadic colorectal cancer using high-resolution melting analysis. *Br J Cancer* **103**, 1627-1636 (2010).

4. Soria, J.C.*, et al.* Lack of PTEN expression in non-small cell lung cancer could be related to promoter methylation. *Clin Cancer Res* **8**, 1178-1184 (2002).
