# Supplementary figures and images for: The natural compound gracillin exerts potent antitumor activity by targeting mitochondrial complex II
Source: Cell Death Dis. 2019 Oct 24;10(11):810. doi: 10.1038/s41419-019-2041-z (PMC6813327; doi:10.1038/s41419-019-2041-z)

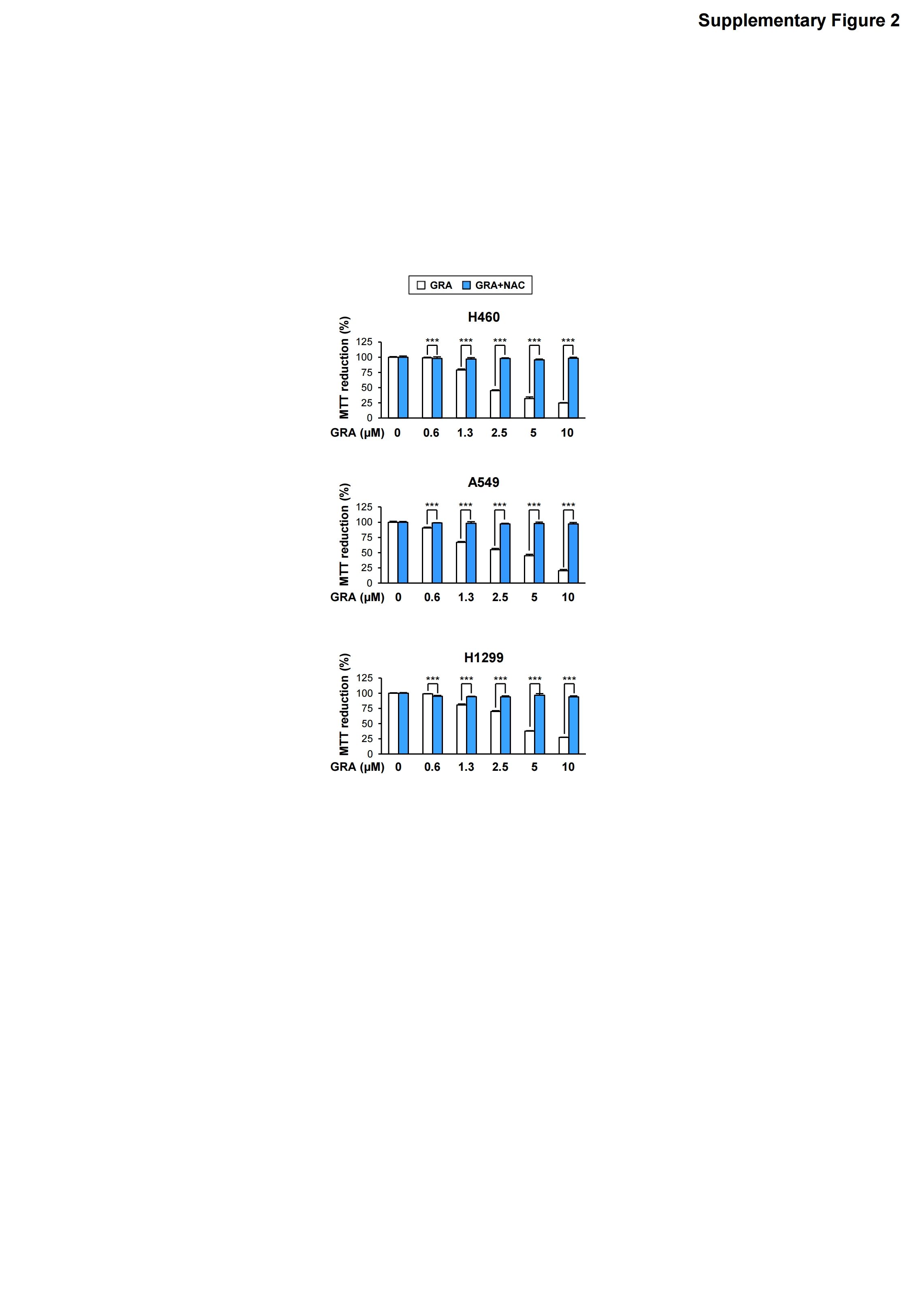

Supplement: Supplementary file 2 — Supplementary Figure legends No further amendments required [file 41419_2019_2041_MOESM2_ESM.png]

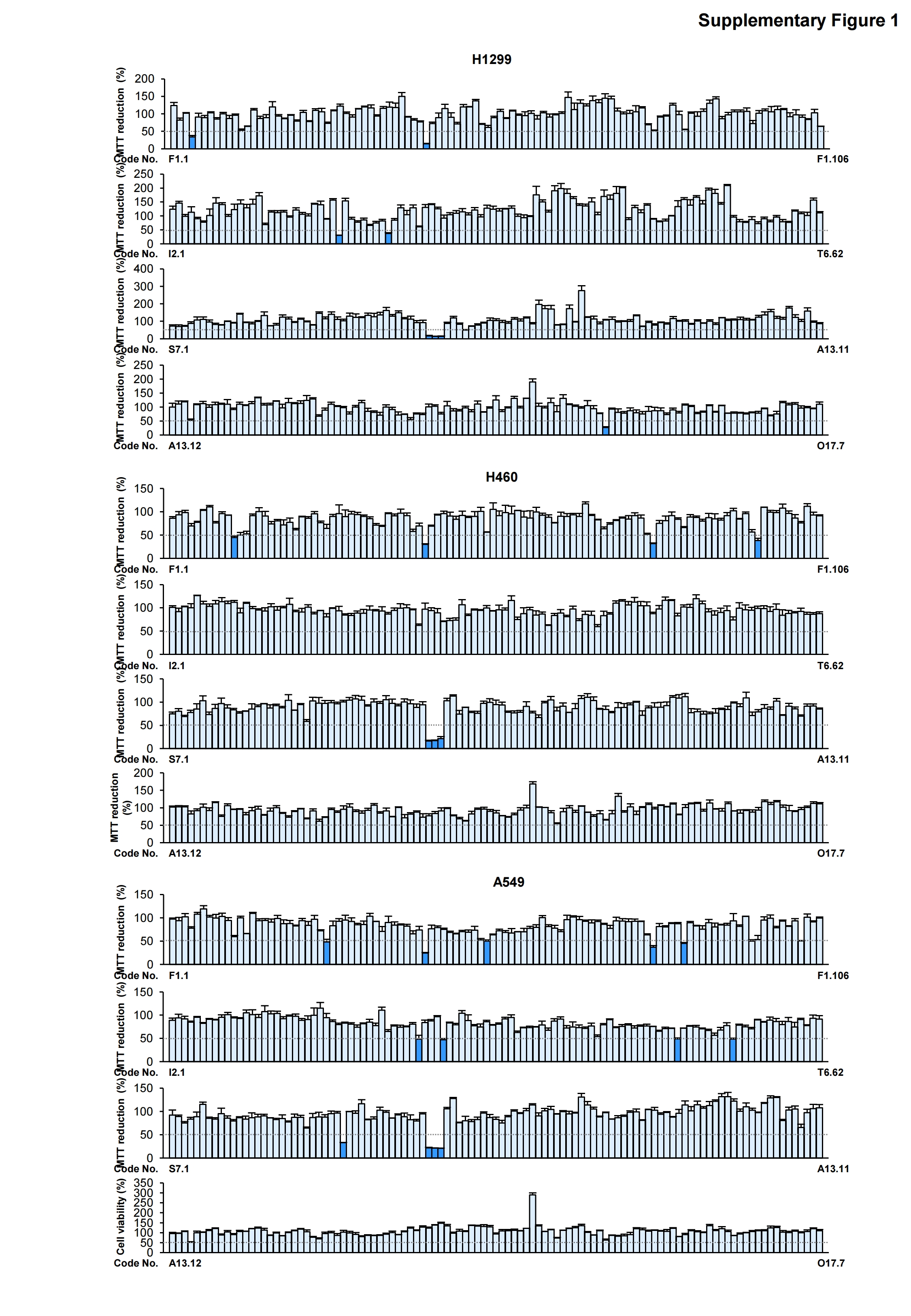

Supplement: Supplementary file 3 — Supplementary Figure 1 No further amendments required [file 41419_2019_2041_MOESM3_ESM.png]

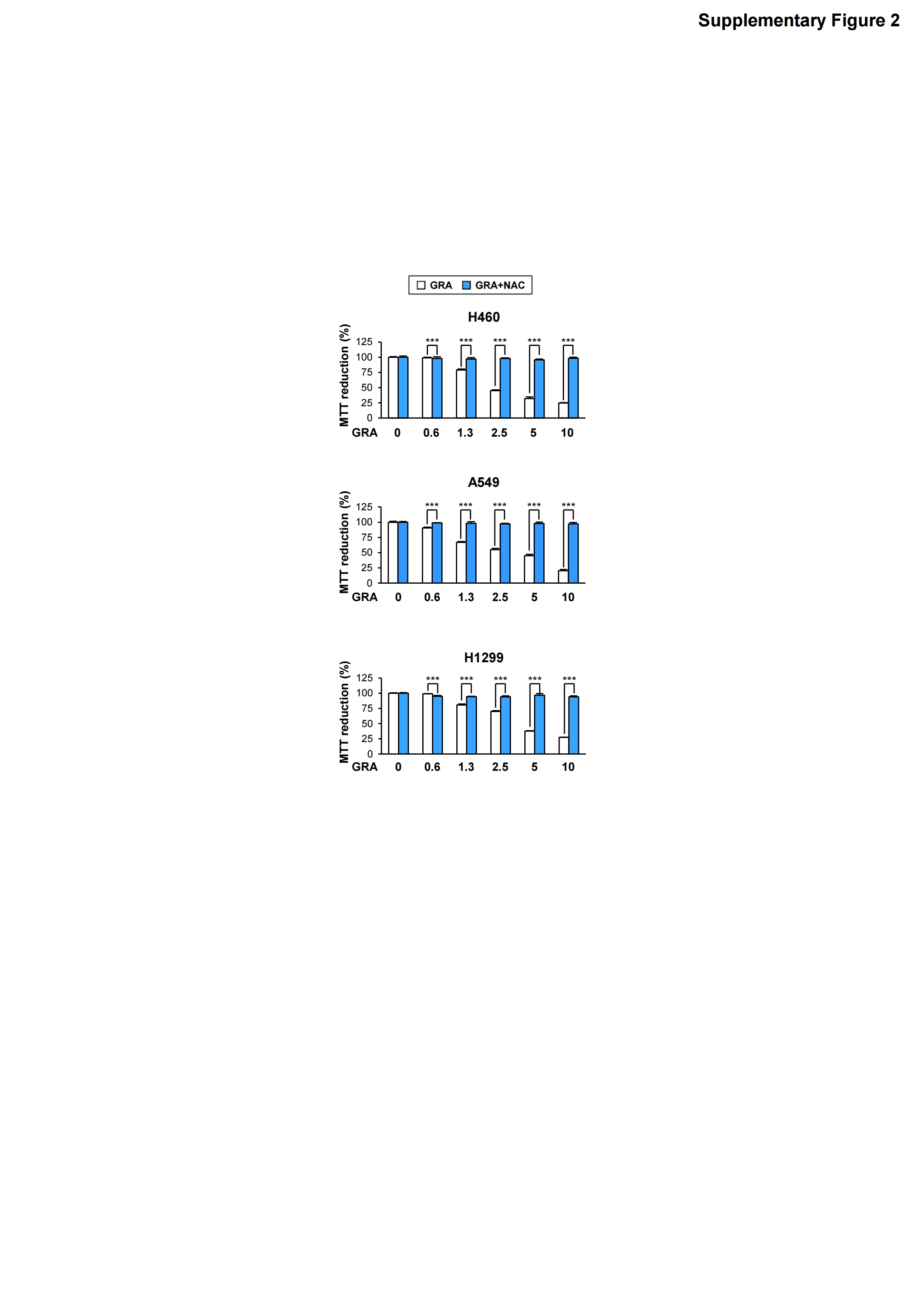

Supplement: Supplementary file 4 — Supplementary Figure 2 The label ‘μM' is missing from the x axis. Please check the attached file (Revised Suppl Fig 2). Thank you. [file 41419_2019_2041_MOESM4_ESM.png]

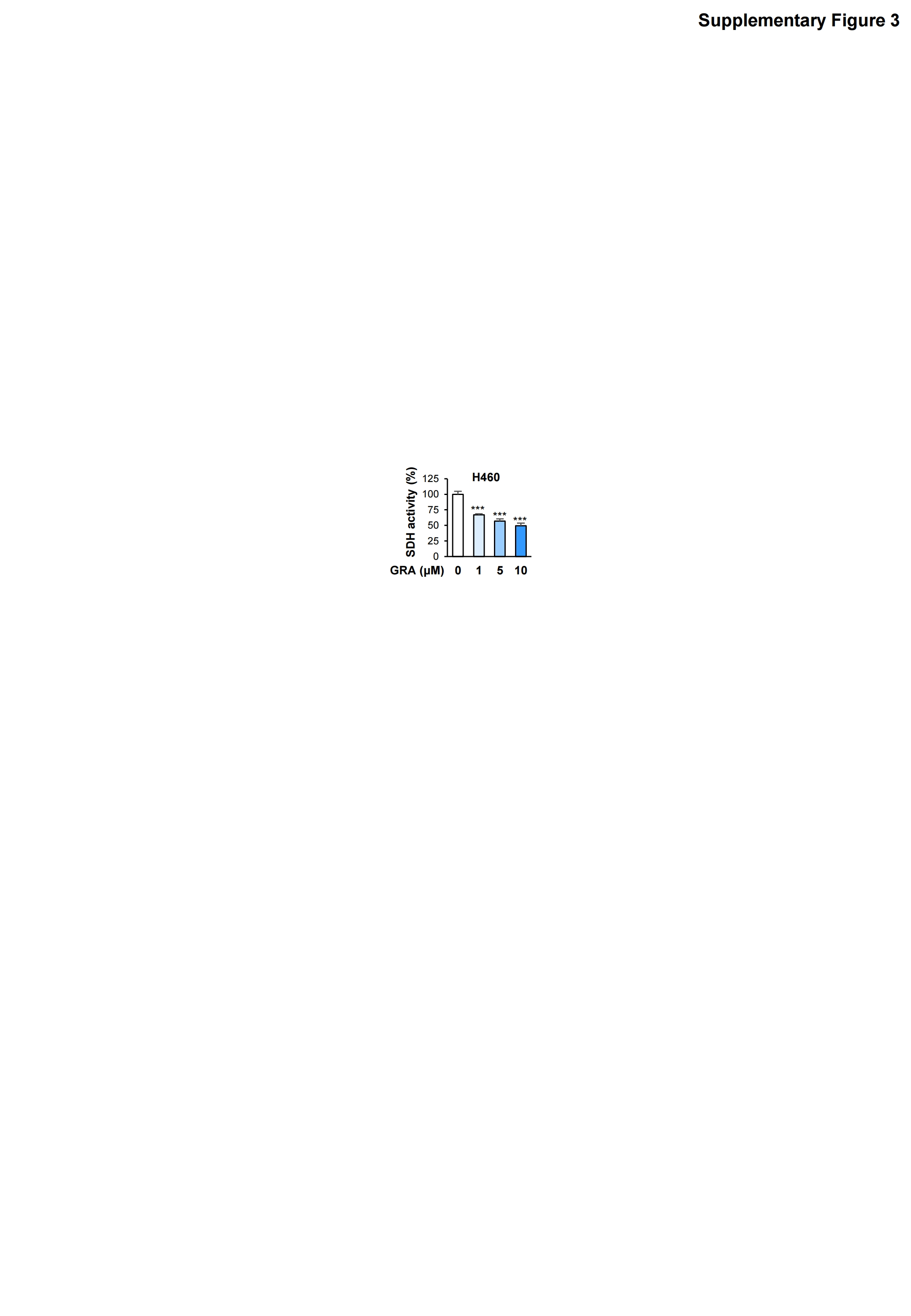

Supplement: Supplementary file 5 — Supplementary Figure 3 No further amendments required [file 41419_2019_2041_MOESM5_ESM.png]

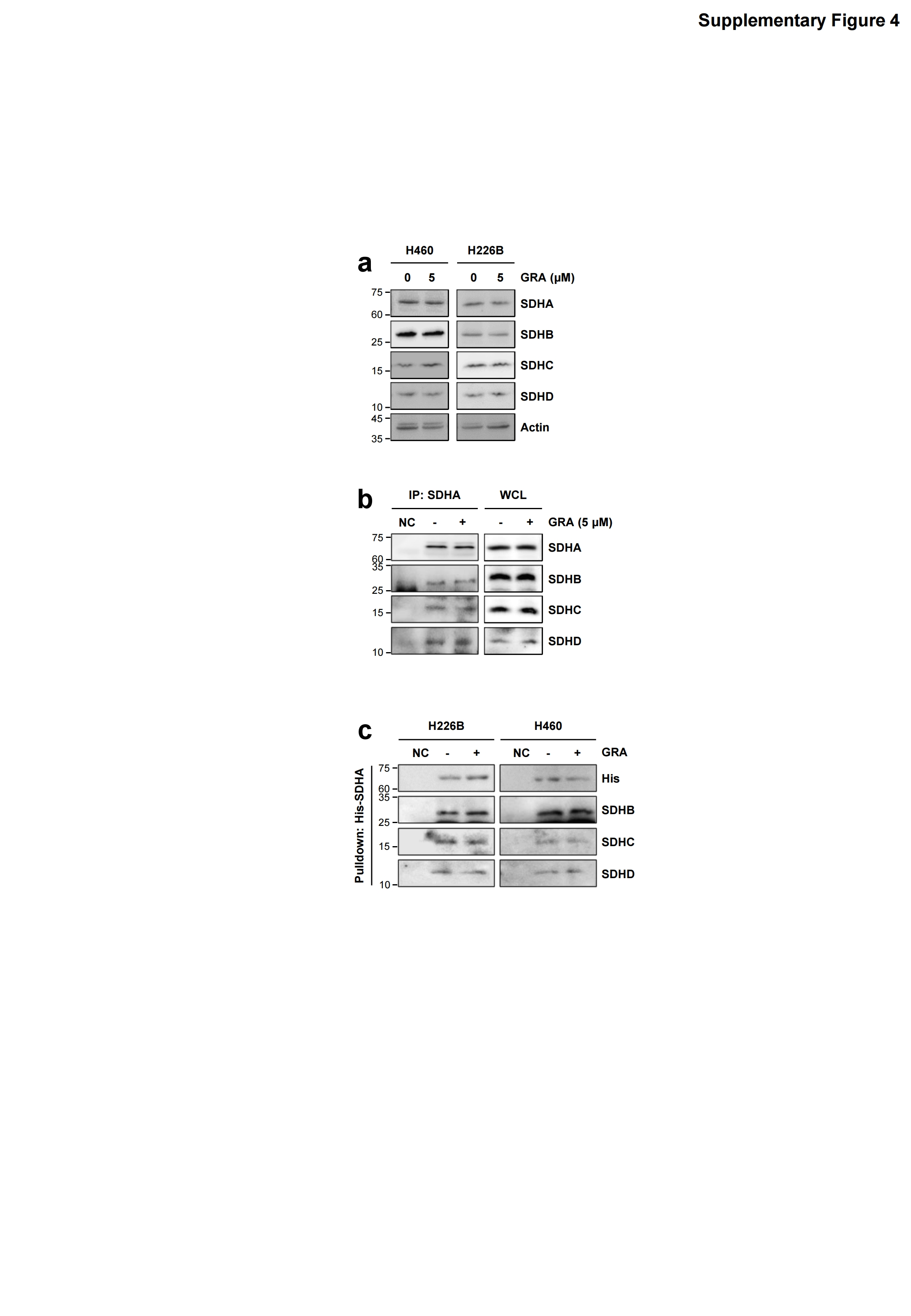

Supplement: Supplementary file 6 — Supplementary Figure 4 No further amendments required [file 41419_2019_2041_MOESM6_ESM.png]
