# Supplementary material for: The natural compound gracillin exerts potent antitumor activity by targeting mitochondrial complex II
Source: Cell Death Dis. 2019 Oct 24;10(11):810. doi: 10.1038/s41419-019-2041-z (PMC6813327; doi:10.1038/s41419-019-2041-z)
Supplement: Supplementary file 7 — Contribution form No further amendments required [file 41419_2019_2041_MOESM7_ESM.pdf]

# DECLARATION OF CONTRIBUTIONS TO ARTICLE

ADMC

Manuscript Number: CDDIS-19-1896RR

Journal Name: *Cell Death & Differentiation* (the 'Journal')

Proposed Title of the Contribution:

The natural compound gracillin exerts potent antitumor activity by targeting mitochondrial complex II (the 'Contribution)

Author(s):

Hye-Young Min, Hyun-Ji Jang, Kwan Hee Park, Seung Yeob Hyun, So Jung Park, Ji Hye Kim, Jaekyoung Son, Sam Sik Kang, Ho-Young Lee (the 'Authors')

For all *CDD* articles, each person named as an author in the published version must be able to show he or she has contributed substantially to the article.

Authorship credit should be based on 1) substantial contributions to conception and design, acquisition of data, or analysis and interpretation of data; 2) drafting the article or revising it critically for important intellectual content; and 3) final approval of the version to be published. Authors should meet conditions 1, 2 and 3.

Any person who cannot be shown to have made a substantial contribution to the article cannot be listed as an author in the final version. The name of any person who is deemed to have made a minor contribution can, however, appear in the Acknowledgments section of the article.

Please complete the table below to indicate the contributions of all named authors to the manuscript.

| Author Full Name: | Specification of Contribution to the Manuscript: |
|---|---|
| Hye-Young Min | acquisition, analysis, and interpretation of data (performing in vitro experiments); drafting the manuscript; final approval of the version to be published |
| Hyun-Ji Jang | acquisition, analysis, and interpretation of data (performing in vitro experiments); final approval of the version to be published |
| Kwan Hee Park | acquisition, analysis, and interpretation of data (performing in vitro and in vivo experiments); final approval of the version to be published |
| Seung Yeob Hyun | acquisition of data (performing in vivo experiments); final approval of the version to be published |
| So Jung Park | acquisition of data (performing in vitro experiments); final approval of the version to be published |
| Ji Hye Kim | acquisition of data (performing LC/MS analysis of metabolites); final approval of the version to be published |
| Jaekyoung Son | analysis and interpretation of data (LC/MS analysis of metabolites); final approval of the version to be published |
| Sam Sik Kang | acquisition of data (providing natural product chemical library including gracillin); final approval of the version to be published |
| Ho-Young Lee | conception and design; analysis and interpretation of data; revising the draft of manuscript; final approval of the version to be published |
| - | - |
| - | - |
| - | - |
| - | - |

Please complete the table below to indicate the contributions of all named authors to the figures.

Figure 1:

Hye-Young Min: acquisition, analysis, and interpretation of data (Fig.1b)
Hyun-Ji Jang: acquisition, analysis, and interpretation of data (Fig.1c-1f)
Kwan Hee Park: none
Seung Yeob Hyun: none
So Jung Park: none
Ji Hye Kim: none
Jaekyoung Son: none
Sam Sik Kang: providing natural product chemical library (Fig 1a-1f)
Ho-Young Lee: conception and design; analysis and interpretation of data

Figure 2:

Hye-Young Min: none
Hyun-Ji Jang: acquisition, analysis, and interpretation of data (Fig. 2a-2i)
Kwan Hee Park: acquisition, analysis, and interpretation of data (Fig. 2a-2f)
Seung Yeob Hyun: none
So Jung Park: none
Ji Hye Kim: none
Jaekyoung Son: none
Sam Sik Kang: none
Ho-Young Lee: conception and design; analysis and interpretation of data

Figure 3:

Hye-Young Min: acquisition, analysis, and interpretation of data (Fig. 3a-3c)
Hyun-Ji Jang: none
Kwan Hee Park: acquisition, analysis, and interpretation of data (Fig. 3a-3c)
Seung Yeob Hyun: none
So Jung Park: none
Ji Hye Kim: none
Jaekyoung Son: none
Sam Sik Kang: none
Ho-Young Lee: conception and design; analysis and interpretation of data

Figure 4:

Hye-Young Min: acquisition, analysis, and interpretation of data (Fig. 4a, 4b)
Hyun-Ji Jang: acquisition, analysis, and interpretation of data (Fig. 4c, 4d, 4f)
Kwan Hee Park: acquisition, analysis, and interpretation of data (Fig. 4c, 4e)
Seung Yeob Hyun: none
So Jung Park: none
Ji Hye Kim: none
Jaekyoung Son: none
Sam Sik Kang: none
Ho-Young Lee: conception and design; analysis and interpretation of data

Figure 5:

Hye-Young Min: acquisition, analysis, and interpretation of data (Fig. 5f, 5g, 5i, 5j)
Hyun-Ji Jang: acquisition, analysis, and interpretation of data (Fig. 5a-5d, 5h)
Kwan Hee Park: none
Seung Yeob Hyun: none
So Jung Park: none
Ji Hye Kim: acquisition of data (Fig. 5e)
Jaekyoung Son: analysis, and interpretation of data (Fig. 5e)
Sam Sik Kang: none
Ho-Young Lee: conception and design; analysis and interpretation of data

Figure 6:

Hye-Young Min: acquisition, analysis, and interpretation of data (Fig. 6a, 6b, 6f, 6k)
Hyun-Ji Jang: acquisition, analysis, and interpretation of data (Fig. 6g)
Kwan Hee Park: acquisition, analysis, and interpretation of data (Fig. 6c-6f, 6h-6j)
Seung Yeob Hyun: acquisition of data (Fig. 6c, 6d, 6h)
So Jung Park: acquisition of data (Fig. 6a, 6b)
Ji Hye Kim: none
Jaekyoung Son: none
Sam Sik Kang: none
Ho-Young Lee: conception and design; analysis and interpretation of data

| Signed for and on behalf of the Author(s): | Print Name: | Date: |
|---|---|---|
| Ho-Young Lee | Ho-Young Lee | 10/01/19 |
